# *KTN1-AS1*, a SOX2-mediated lncRNA, activates epithelial–mesenchymal transition process in esophageal squamous cell carcinoma

**DOI:** 10.1038/s41598-022-24743-z

**Published:** 2022-11-23

**Authors:** Liying Chen, Juntao Lu, Tongxin Xu, Zhaoyang Yan, Yanli Guo, Zhiming Dong, Wei Guo

**Affiliations:** Laboratory of Pathology, Hebei Cancer Institute, The Fourth Hospital of Hebei Medical University, Jiankang Road 12, Shijiazhuang, 050011 Hebei China

**Keywords:** Cancer, Oncology

## Abstract

Kinectin 1 antisense RNA 1 (*KTN1-AS1*), a long non-coding RNA (lncRNA), has been proved to have tumor-promoting properties and its expression is enhanced in several human tumors. However, the role of *KTN1-AS1* in the pathogenesis of esophageal squamous cell carcinoma (ESCC) remains unknown. This study aimed to investigate the expression status, functional roles, and molecular mechanisms of *KTN1-AS1* in the development of ESCC. Considerable upregulation of *KTN1-AS1* was confirmed in esophageal cancer cells and ESCC tissues and its expression was associated with TNM stage, pathological differentiation, and lymph node metastasis. SOX2 directly activated transcription of *KTN1-AS1*, and overexpression of *KTN1-AS1* facilitated ESCC cells proliferation and invasion in vitro and in vivo. Furthermore, *KTN1-AS1* could bind to retinoblastoma binding protein 4 (RBBP4) in the nucleus and enhanced its binding with histone deacetylase 1 (HDAC1), thereby activating the epithelial–mesenchymal transition (EMT) process through downregulating *E-cadherin* expression at the epigenetic level. In conclusion, *KTN1-AS1*, induced by SOX2, acts as a tumor-promoting gene and may serve as a potential therapeutic and prognostic biomarker for ESCC.

## Introduction

Esophageal cancer is one of the most common malignant tumors worldwide, ranking sixth and seventh in mortality and morbidity, respectively, among all tumors. Esophageal squamous cell carcinoma (ESCC) is one of the predominant histological types of esophageal cancer^1,2^. The technology for diagnosis and treatment of esophageal cancer has constantly improved^3^. However, owing to the lack of specific symptoms and sensitive screening methods at an early stage, most patients with esophageal cancer are diagnosed at an advanced stage. Moreover, the 5-year survival rate after comprehensive treatment remains below 20% due to recurrence and distant metastasis. Therefore, identification of novel diagnostic and prognostic biomarkers, in addition to sensitive mediators of metastasis and recurrence, is highly desirable for improving the prognosis and survival of ESCC patients.

LncRNAs, with lengths > 200 nucleotides, represent diverse types of RNA molecules with limited or no protein-coding capability, and different biological functions depending on their subcellular location. Most nuclear lncRNAs participate in transcriptional regulation by recruiting DNA methyltransferase or histone acetylation and deacetylation enzymes to specific genomic sites or by interacting with RNA-binding proteins to affect activity of transcription complex^4,5^. Cytoplasmic lncRNAs, excluding their mRNA regulatory function^6^, can affect target gene expression by modulating microRNAs (miRNAs)^7,8^. *KTN1-AS1* is located on human chromosome 14q22.3, and is one of three lncRNA signatures derived from the Atlas of ncRNA in cancer (TANRIC) database for predicting survival of patients with head and neck squamous cell carcinoma^9^. Subsequent studies have shown that *KTN1-AS1* played an important role in the development and progression of non-small cell lung cancer, hepatocellular carcinoma, glioma, pancreatic cancer, bladder cancer, and ovarian cancer^10–16^. However, the role of *KTN1-AS1* in ESCC has not yet been elucidated.

In this study, we detected the expression of *KTN1-AS1* in ESCC tissues and cells, and analyzed the relationship of *KTN1-AS1* with clinical characteristics of ESCC patients. Further studies focused on its functions on ESCC cells in vitro and in vivo, and explored the potential mechanisms of *KTN1-AS1* in the carcinogenesis of ESCC.

## Results

### Upregulated expression of *KTN1-AS1* in ESCC

**Figure 1:**
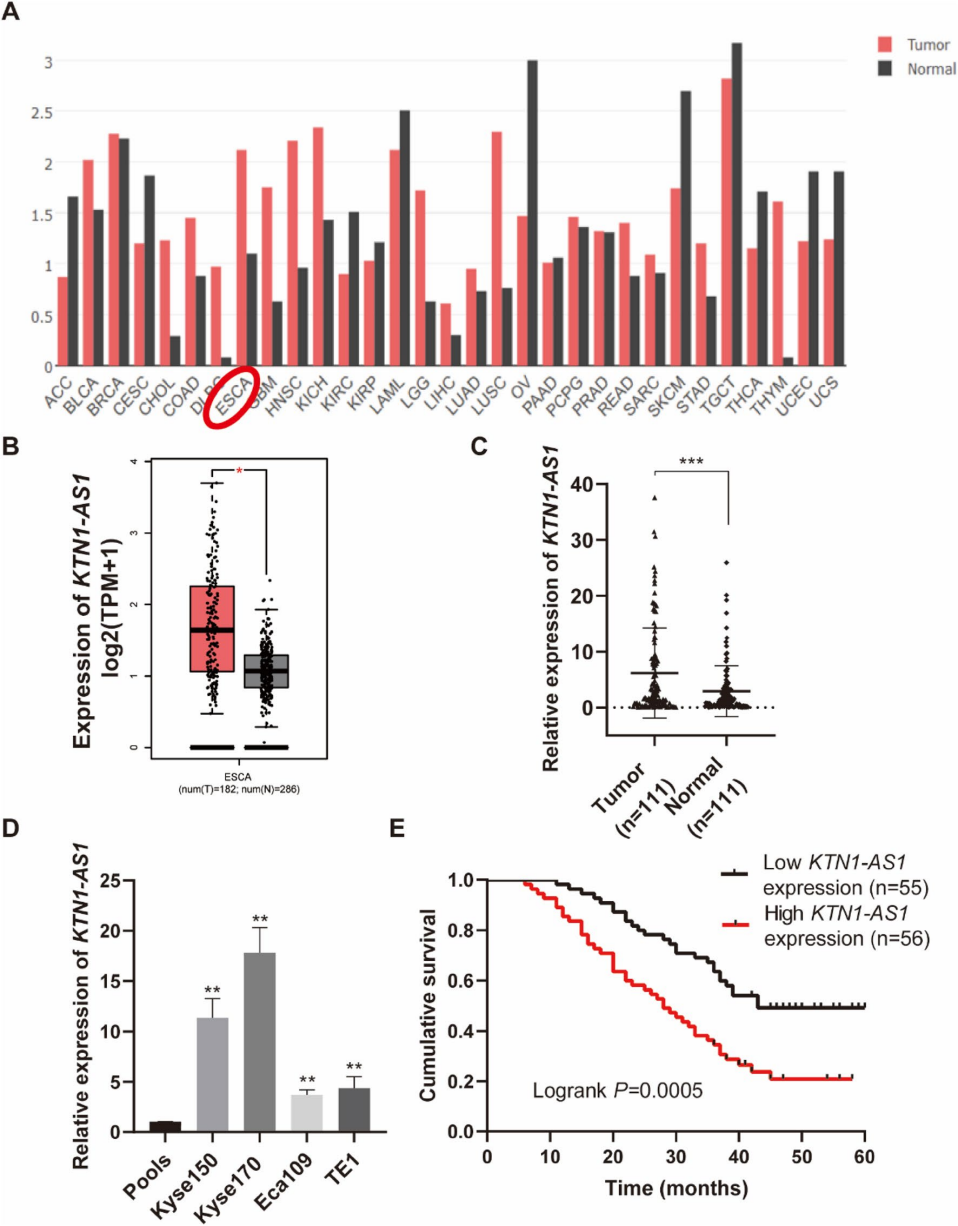
*KTN1-AS1* is upregulate in esophageal squamous cell carcinoma (ESCC) tissues and is associated with poor prognosis. (**A**) The *KTN1-AS1* expression profile across all tumor samples and corresponding normal tissues obtained from the Gene Expression Profiling Interactive Analysis (GEPIA). (**B**) The relative expression of *KTN1-AS1* in 182 tumor samples compared with 286 normal samples obtained from the GEPIA database. (**C**) The expression levels of *KTN1-AS1* in ESCC and the corresponding normal tissues were detected by qRT-PCR method. (**D**) The expression levels of *KTN1-AS1* in human ESCC cell lines [Kyse150, Kyse170, Eca109, TE1, and Pools (the normal control comes from average expression of 10 normal tissues)]. (**E**) The overall survival of 111 patients with ESCC with high or low *KTN1-AS1* expression was assessed using Kaplan Meier analysis; log-rank *P* = 0.0005. Error bars are shown as mean ± SD from three replicate experiments (n = 3) (*P < 0.05, **P < 0.01, ***P < 0.001).

The expression levels of *KTN1-AS1* were higher in many tumors including ESCC according to the Gene Expression Profiling Interactive Analysis (GEPIA) database (Fig. 1A,B). The elevated expression of *KTN1-AS1* was also detected in ESCC tissues and human esophageal cancer cell lines (Fig. 1C,D). Furthermore, based on the median expression value of *KTN1-AS1*, patients with ESCC were divided into high and low expression groups (n = 56 and n = 55, respectively); and *KTN1-AS1* expression was found to be related to TNM stage, pathological differentiation, and lymph node metastasis (Table 1). Moreover, *KTN1-AS1* expression was associated with ESCC patients’ survival (Fig. 1E). Results of univariate and multivariate Cox regression analysis suggested that depth of invasion, lymph node metastasis, and expression level of *KTN1-AS1* were independent prognostic indicators for ESCC patients (Table 2).

**Table 1:** Relative expression level of *KTN1-AS1* in ESCC patients.

Characteristics	N	*KTN1-AS1* expression		*P* value
		Low n (%)	High n (%)	
**Age (years)**				0.397
< 60	46	25 (54.3%)	21 (45.7%)	
≥ 60	65	30 (46.2%)	35 (53.8%)	
**Gender**				0.138
Male	69	38 (55.1%)	31 (44.9%)	
Female	42	17 (40.5%)	25 (59.5%)	
**Smoking**				0.782
Negative	51	26 (51.0%)	25 (49.0%)	
Positive	60	29 (48.3%)	31 (51.7%)	
**Family history of UGIC**				0.442
Negative	90	43 (47.8%)	47 (52.2%)	
Positive	21	12 (57.1%)	9 (42.9%)	
**TNM stage**				< 0.001
I + II	39	33 (84.6%)	6 (15.4%)	
III + IV	72	22 (30.6%)	50 (69.4%)	
**Depth of invasion**				0.633
T1/2	58	30 (51.7%)	28 (48.3%)	
T3/4	53	25 (47.2%)	28 (52.8%)	
**Pathological differentiation**				< 0.001
Well/moderate	64	49 (76.6%)	15 (23.4%)	
Poor	47	6 (12.8%)	41 (87.2%)	
**LN metastasis**				< 0.001
Negative (N0)	32	25 (78.1%)	7 (21.9%)	
Positive (N1/2/3)	79	30 (38.0%)	49 (62.0%)

**Table 2:** Univariate and multivariate Cox regression analysis for clinicopathological features with prognosis of patients with ESCC.

Variables	Univariate analysis	*P*	Multivariate analysis	*P*
	HR (95% CI)		HR (95% CI)	
Age (< 60 vs. ≥ 60)	1.407 (0.865–2.287)	0.168	0.794 (0.472–1.334)	0.383
Gender (male vs. female)	0.812 (0.493–1.338)	0.415	0.831 (0.446–1.550)	0.561
Smoking (negative vs. positive)	1.086 (0.676–1.745)	0.732	0.883 (0.486–1.606)	0.683
Family history of UGIC (negative vs. positive)	0.620 (0.317–1.212)	0.162	0.876 (0.423–1.816)	0.722
TNM stage (I/II vs. III/IV)	3.086 (1.737–5.484)	< 0.001	0.800 (0.308–2.076)	0.646
Depth of invasion (T1/2 vs. T3/4)	2.895 (1.781–4.706)	< 0.001	2.928 (1.798–4.769)	< 0.001
LN metastasis (N0 vs. N1/2/3)	3.434 (1.798–6.557)	< 0.001	2.703 (1.381–5.291)	0.004
Pathological differentiation (well/moderate vs. poor)	2.232 (1.391–3.580)	0.001	1.046 (0.526–2.083)	0.897
*KTN1-AS1* (low vs. high)	2.354 (1.449–3.823)	0.001	1.893 (1.145–3.131)	0.013

### SOX2 induces *KTN1-AS1* expression in ESCC cells

**Figure 2:**
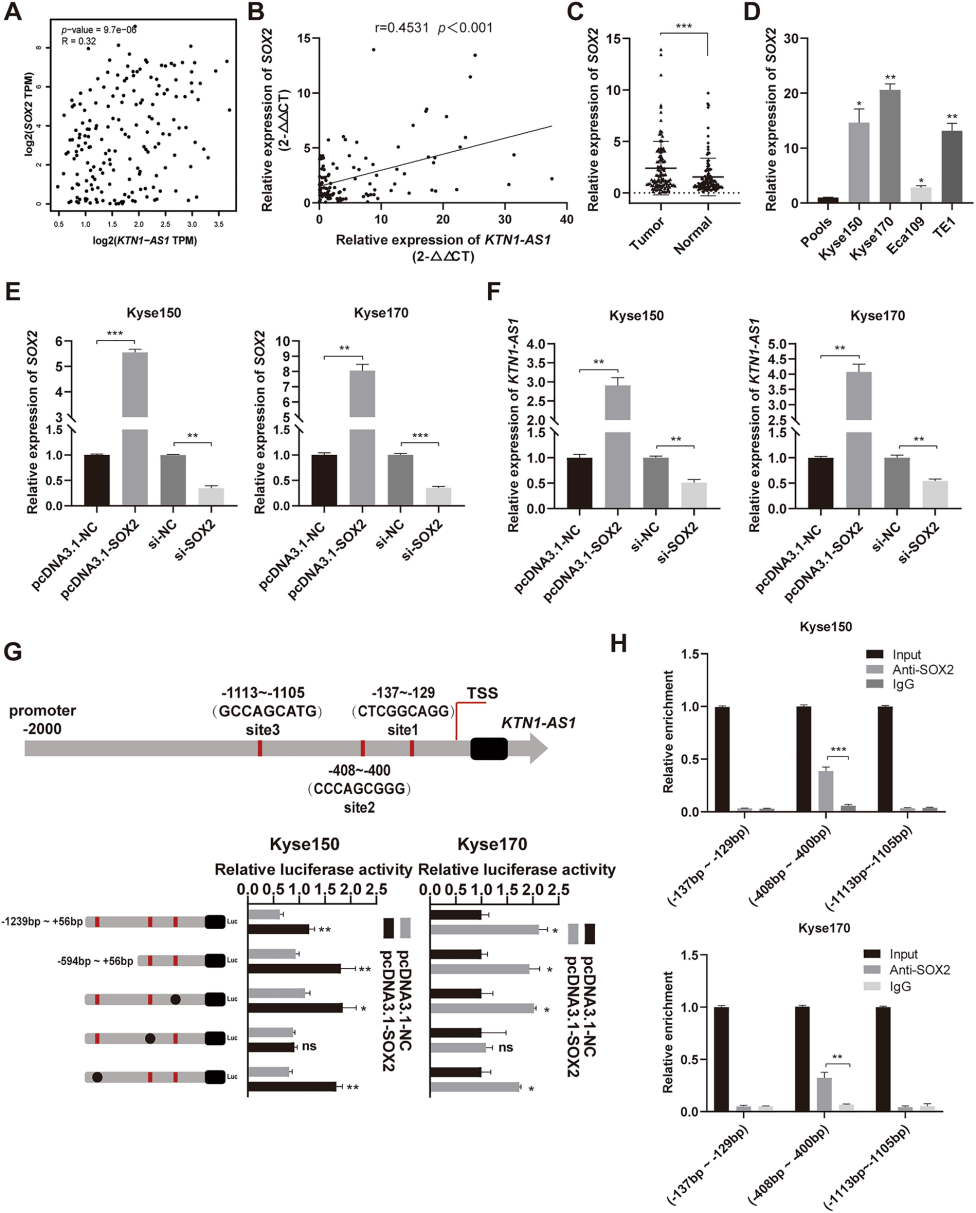
SOX2 induces *KTN1-AS1* expression in esophageal squamous cell carcinoma (ESCC) cells. (**A**) The relevance of expression between *KTN1-AS1* and *SOX2* was analyzed by the Gene Expression Profiling Interactive Analysis (GEPIA) database. (**B**) The correlation between the expression of *KTN1-AS1* and *SOX2* was analyzed by qRT-PCR method. (**C**) The expression levels of *SOX2* in ESCC and the corresponding normal tissues were detected by qRT-PCR method. (**D**) The expression levels of *SOX2* in human ESCC cell lines. (**E**) The overexpression and knockdown efficiency of *SOX2* in Kyse150 and Kyse170 cells were detected by qRT-PCR method. (**F**) The regulation effect of *SOX2* on *KTN1-AS1* expression in Kyse150 and Kyse170 cells were detected using qRT-PCR method. (**G**) Luciferase activity assay was conducted in Kyse150 and Kyse170 cells to verify the direct binding of SOX2 to the *KTN1-AS1* promoter. (**H**) Chromatin Immunoprecipitation (ChIP) assay was used to confirm the binding site of SOX2 on the promoter of *KTN1-AS1* in Kyse150 and Kyse170 cells. Error bars are shown as mean ± SD from three replicate experiments (n = 3) (*P < 0.05, **P < 0.01, ***P < 0.001).

Given the high expression of *KTN1-AS1* in ESCC, the mechanism leading to its upregulation attracted our attention. To study the potential transcription factors regulating *KTN1-AS1*, we searched two online databases, hTFtarget and animalTFDB3, and predicted that there were 127 transcription factors (Supplementary Fig. S1A). We further analyzed the correlation between the 127 transcription factors and the expression of *KTN1-AS1* through the GEPIA database, and found that there were 36 transcription factors with an R value greater than 0.3, including SOX2 (Supplementary Fig. S1B). A positive correlation was found between *SOX2* and *KTN1-AS1* (Fig. 2A,B) and the mRNA expression of *SOX2* was also significantly upregulated in ESCC tissues and cell lines (Fig. 2C,D). After transfection of pcDNA3.1-SOX2 and si-SOX2 into Kyse150 and Kyse170 cells (Fig. 2E), the expression level of *KTN1-AS1* was substantially increased in *SOX2* overexpressing cells and remarkably decreased in *SOX2* knockdown cells (Fig. 2F). So we chose SOX2 for the following study.

As shown in Fig. 2G, according to the location of the three possible binding sites (site 1: − 137 bp to − 129 bp, site 2: − 408 bp to − 400 bp, and site 3: − 1113 to − 1105 bp), we constructed a luciferase reporter gene plasmid containing the − 1239 bp to + 56 bp region of *KTN1-AS1* promoter, and co-transfected it with pcDNA3.1-SOX2 in Kyse150 and Kyse170 cells, respectively. The luciferase activity was found to be substantially increased. To further clarify the specific site of action, a truncated plasmid containing the fragment of − 594 bp to + 56 bp was constructed, and its luciferase activity was also considerably upregulated. The elevated luciferase activity was significantly decreased when site 2 was mutated, while no obvious change was observed accompanied with site 1 or site 3 mutation, suggesting the key role of site 2. The binding effect of SOX2 on site 2 of the *KTN1-AS1* promoter was further confirmed by ChIP assay (Fig. 2H). These results collectively suggest that the elevated expression of *KTN1-AS1* may be regulated by SOX2 in ESCC.

### *KTN1-AS1* facilitates proliferation, migration, and invasion of ESCC cells

**Figure 3:**
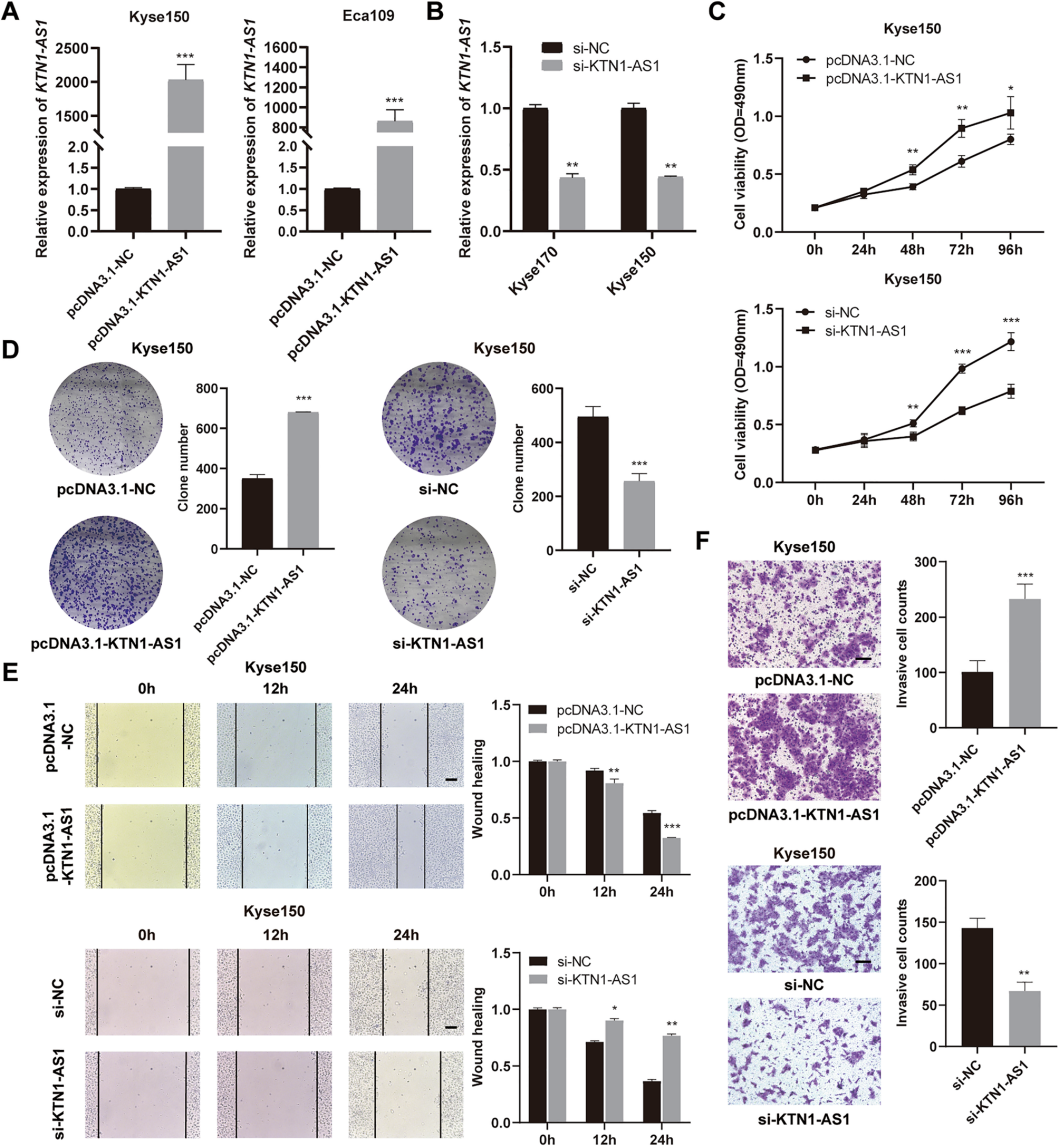
*KTN1-AS1* facilitates esophageal squamous cell carcinoma (ESCC) cells biological functions. (**A**,**B**) The overexpression efficiency of *KTN1-AS1* in Kyse150 and Eca109 cells, and the knockdown efficiency of *KTN1-AS1* in Kyse170 and Kyse150 cells. (**C**,**D**) MTS and clone formation assays were used to demonstrate the cell proliferation ability in Kyse150 cells in response to upregulation and downregulation of *KTN1-AS1*. (**E**,**F**) Cell migration and invasion ability were verified through wound healing and transwell assays in Kyse150 cells by overexpressing and downregulating *KTN1-AS1* (magnification: ×100). Error bars are shown as mean ± SD from three replicate experiments (n = 3) (*P < 0.05, **P < 0.01, ***P < 0.001).

To investigate the biological function of *KTN1-AS1* in ESCC cells, the overexpression plasmid of *KTN1-AS1* was transfected into Kyse150 and Eca109 cells, and a striking upregulation of *KTN1-AS1* was detected in the transfected cells (Fig. 3A). Furthermore, si-KTN1-AS1 was used to knockdown the expression of *KTN1-AS1* in Kyse170 and Kyse150 cells (Fig. 3B). Overexpression of *KTN1-AS1* stimulated the proliferation, migration, and invasion capability of Kyse150 cells, similar results were obtained in *KTN1-AS1* overexpressed Eca109 cells (Supplementary Fig. S2A–D). While knockdown of *KTN1-AS1* alleviated the growth, migration, and invasion ability of Kyse150 (Fig. 3C–F) and Kyse170 cells (Supplementary Fig. S2E–H). These results suggest that *KTN1-AS1* may play an oncogenic role in ESCC.

### *KTN1-AS1* interacts with RBBP4 in the nucleus

**Figure 4:**
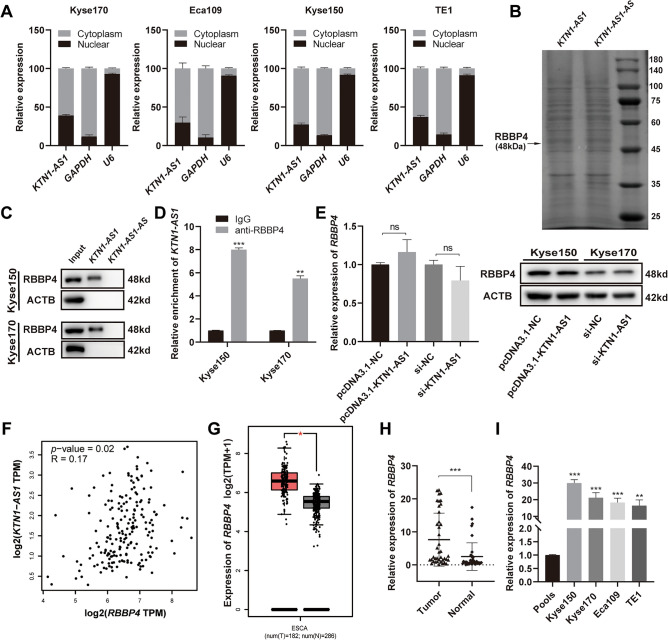
*KTN1-AS1* interacts with RBBP4 in the nucleus. (**A**) The subcellular location of *KTN1-AS1* in esophageal squamous cell carcinoma (ESCC) cell lines. (**B**) RNA pull-down assay was performed in Kyse150 and Kyse170 cells, and the RNA-related proteins were determined with SDS-PAGE gel and coomassie brilliant blue staining. Original gel image was presented in Supplementary Fig. S5A. (**C**) Western blot assay was performed to detect the specific association between RBBP4 and *KTN1-AS1* in Kyse150 and Kyse170 cells. Original western blots were presented in Supplementary Fig. S5B, with blots cut prior to hybridization with antibodies. (**D**) RIP assay showed the interaction between *KTN1-AS1* and RBBP4 in Kyse150 and Kyse170 cells. (**E**) The regulation effect of *KTN1-AS1* on RBBP4 expression was detected by qRT-PCR and western blot. Original western blots were presented in Supplementary Fig. S5C, with blots cut prior to hybridization with antibodies. (**F**) The relevance between *KTN1-AS1* and *RBBP4* expression was predicted by the GEPIA database. (**G**) The relative expression of *RBBP4* in 182 tumor samples compared with 286 normal samples obtained from the Gene Expression Profiling Interactive Analysis (GEPIA) database. (**H**) The expression levels of *RBBP4* in ESCC and the corresponding normal tissues. (**I**) The expression levels of *RBBP4* in ESCC cell lines. Error bars are shown as mean ± SD from three replicate experiments (n = 3) (*P < 0.05, **P < 0.01, ***P < 0.001).

The subcellular localization analysis in ESCC cells showed that *KTN1-AS1* was distributed in both nucleus and cytoplasm (Fig. 4A). RNA pull-down assay followed by mass spectrometry analysis was then performed to detect the RNA binding proteins, and RBBP4 was proved to be one of the differentially expressed proteins (Fig. 4B). The interaction between *KTN1-AS1* and RBBP4 was further verified by Western blot and RIP assay (Fig. 4C,D). However, there were no significant differences in *RBBP4* mRNA and protein expression levels in *KTN1-AS1* overexpressed or knockdown cells (Fig. 4E), which was consistent with the poor correlation predicted by the GEPIA database (Fig. 4F). The expression level of *RBBP4* was upregulated in esophageal carcinoma according to GEPIA database (Fig. 4G). Our results also showed an upregulation of *RBBP4* in ESCC tissues and cell lines (Fig. 4H,I).

### *KTN1-AS1* relates to the epithelial-to-mesenchymal transition (EMT) process by inhibiting the expression of E-cadherin at the epigenetic level

**Figure 5:**
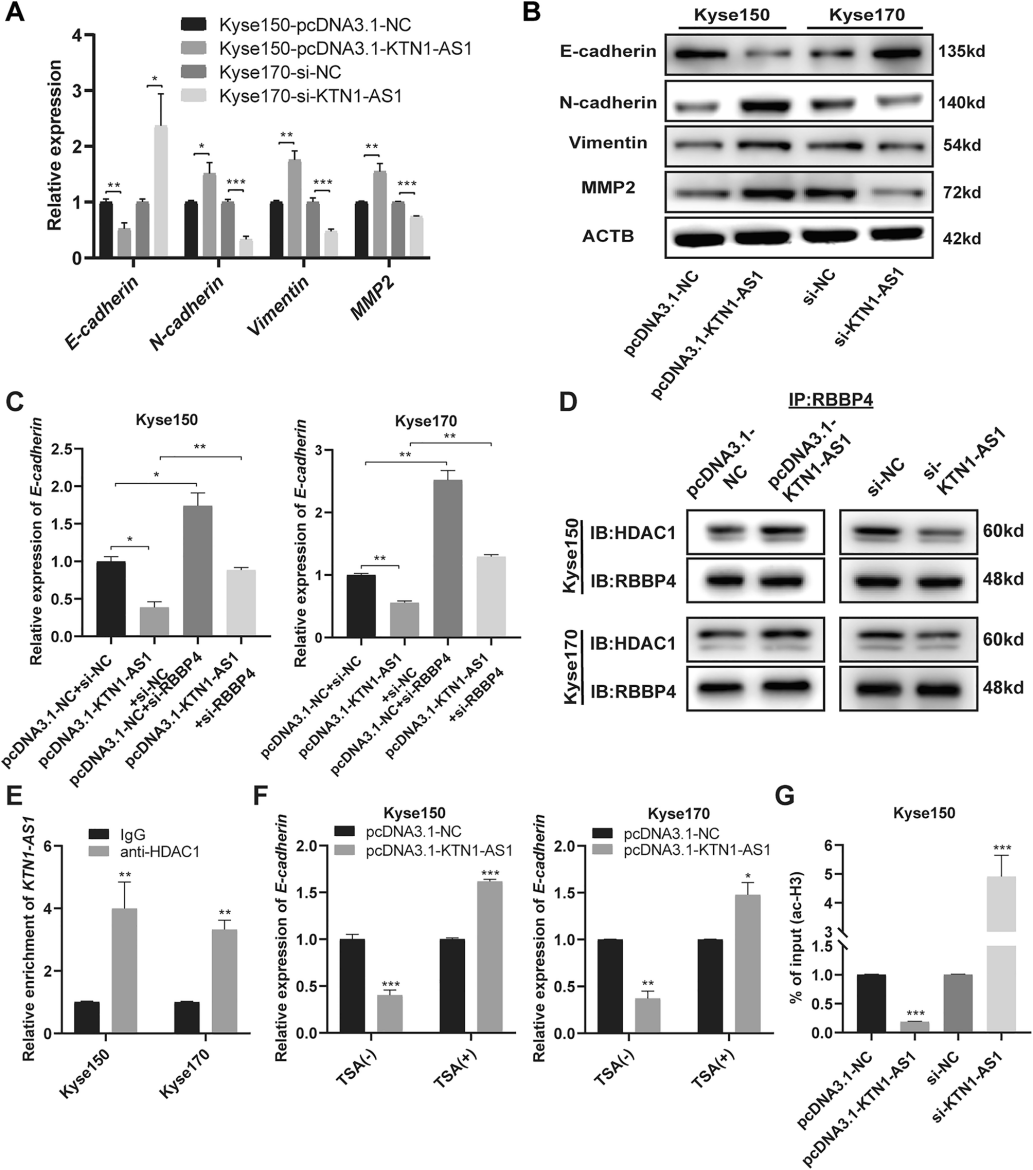
*KTN1-AS1* relates to epithelial-to-mesenchymal transition (EMT) process by interacting with RBBP4 and HDAC1 to silence *E-cadherin* expression. (**A**) The mRNA expression levels of *E-cadherin*, *N-cadherin*, *Vimentin*, and *MMP2* after *KTN1-AS1* overexpression and knockdown. (**B**) The regulatory effect of *KTN1-AS1* on protein levels of E-cadherin, N-cadherin, Vimentin, and MMP2 was detected by western blot. Original western blots were presented in Supplementary Fig. S5D, with blots cut prior to hybridization with antibodies. (**C**) Inhibition of *RBBP4* increased the expression level of *E-cadherin* and partially reversed the regulation effect of *KTN1-AS1* on the expression level of *E-cadherin* in Kyse150 and Kyse170 cells. (**D**) Co-IP assay was performed to examine the RBBP4-HDAC1 interaction in the groups with *KTN1-AS1* overexpression and inhibition in Kyse150 and Kyse170 cells. Original western blots were presented in Supplementary Fig. S5E, with blots cut prior to hybridization with antibodies. (**E**) RIP assay showed the interaction between *KTN1-AS1* and HDAC1 in Kyse150 and Kyse170 cells. (**F**) After transfection of pcDNA3.1-NC or pcDNA3.1-KTN1-AS1 for 12–24 h in Kyse150 and Kyse170 cells, then cells with pcDNA3.1-KTN1-AS1 were treated with or without 300 nM Trichostatin A (TSA) for additional 48 h, the mRNA expression of *E-cadherin* was detected by qRT-PCR method. (**G**) ChIP-qPCR was performed to detect the enrichment of ac-H3 in the promoter region of *E-cadherin* after overexpression and knockdown of *KTN1-AS1* in Kyse150 cells. Error bars are shown as mean ± SD from three replicate experiments (n = 3) (*P < 0.05, **P < 0.01, ***P < 0.001).

Considering that *SOX2* could influence the migration and invasion capability of esophageal cancer cells and up-regulate the expression of *KTN1-AS1* at transcriptional level, we then detected the influence of *KTN1-AS1* on EMT related markers. As shown in Fig. 5A,B, overexpression of *KTN1-AS1* in Kyse150 cells considerably decreased the mRNA and protein expression levels of *E-cadherin*, and increased those of *N-cadherin*, *vimentin*, and *MMP2;* while downregulation of *KTN1-AS1* in Kyse170 cells demonstrated the opposite tendency, suggesting the possible role of *KTN1-AS1* in EMT process. Subsequently, we explored the expression changes of some EMT related markers, including *E-cadherin, N-cadherin*, *Vimentin*, *MMP2*, *Snail1*, and *Twist1*, after knocking down RBBP4. With the downregulation of RBBP4 in Kyse170 cells, the mRNA expression level of *MMP2* was correspondingly downregulated, while the expression of *E-cadherin* was upregulated (Supplementary Fig. S3).

As previous studies have demonstrated the involvement of HDAC1 in the transcriptional regulation of *E-cadherin*^17,18^, and RBBP4 and HDAC1/2 have been proved to form a core deacetylase complex in both the NuRD and Sin3 complex^19,20^, we proposed that *KTN1-AS1* might regulate *E-cadherin* expression at the epigenetic level in the nucleus. The expression of *E-cadherin* was increased after knocking down of *RBBP4*, and the inhibition of *E-cadherin* due to overexpression of *KTN1-AS1* could be alleviated by the inhibition of *RBBP4* (Fig. 5C). The interaction between RBBP4 and HDAC1 was noticeably strengthened in *KTN1-AS1* overexpressed cells, while impaired in *KTN1-AS1* knockdown cells detected by Co-IP assay (Fig. 5D), furthermore, *KTN1-AS1* was also verified to bind with HDAC1 (Fig. 5E), suggesting that *KTN1-AS1* could combine with RBBP4 and HDAC1 to form a complex and simultaneously enhanced the binding effect of RBBP4 and HDAC1. In addition, a rescue experiment was conducted using the HDAC1 inhibitor TSA. As shown in Fig. 5F, TSA treatment alleviated the downregulation of *E-cadherin* caused by overexpression of *KTN1-AS1*. Moreover, as shown in Fig. 5G, overexpression of *KTN1-AS1* weakened the enrichment of acetylation of histone H3 (ac-H3) at the promoter region of *E-cadherin*, whereas knockdown of *KTN1-AS1* enhanced the enrichment of ac-H3, indicating that the binding action of *KTN1-AS1* with RBBP4 and HDAC1 could finally influence the expression of *E-cadherin* via regulating the level of histone acetylation.

### Inhibition of RBBP4 partially reverses the promoting effect of *KTN1-AS1* on the biological behavior of ESCC cells

**Figure 6:**
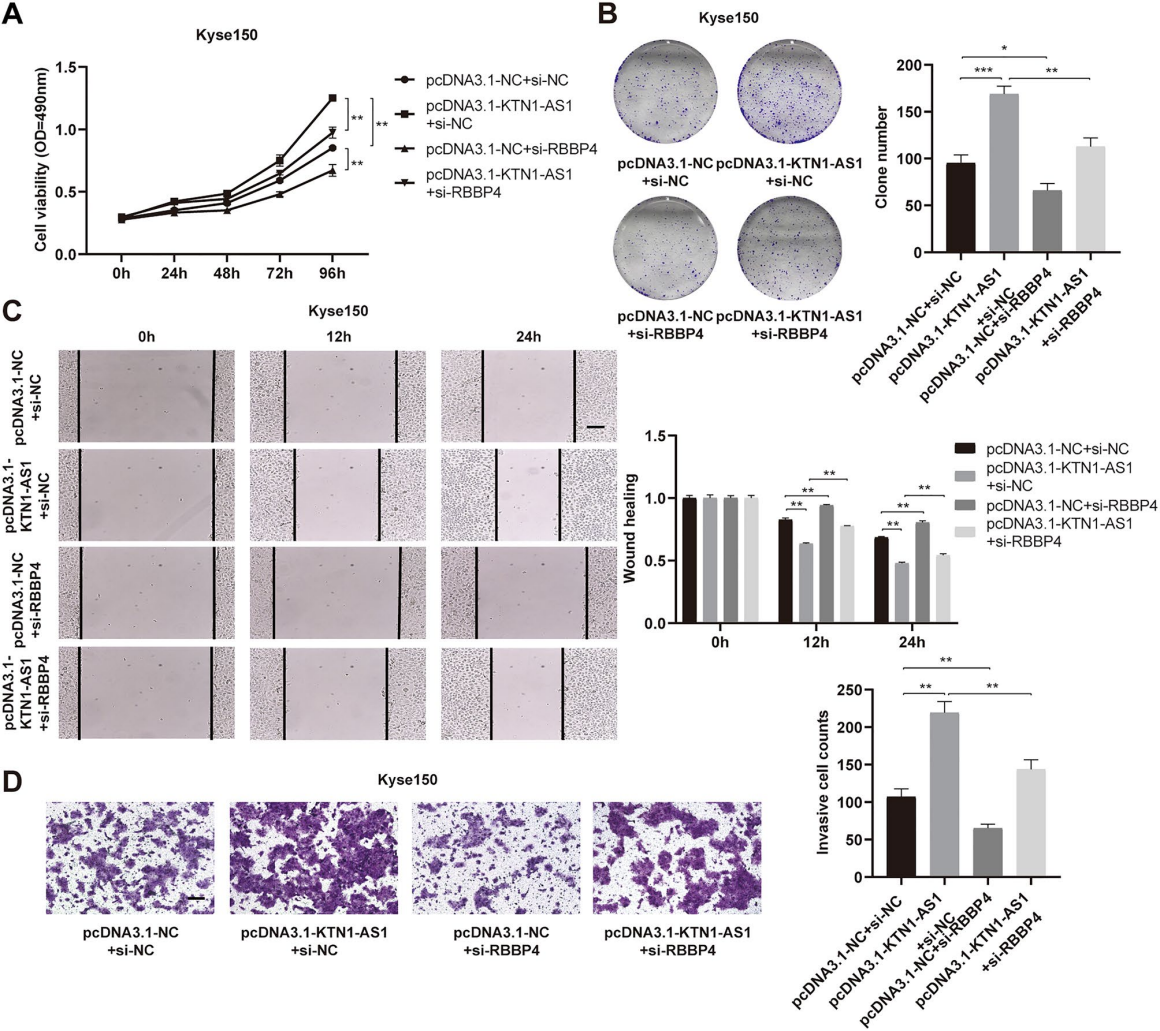
RBBP4 partially reverses the biological function of *KTN1-AS1* on esophageal squamous cell carcinoma (ESCC) cells. (**A**,**B**) MTS and clone formation assays were performed to analyze the cell proliferation ability after co-transfected with pcDNA3.1-KTN1-AS1 and si-RBBP4 in Kyse150 cells. (**C**,**D**) Wound healing and transwell invasion assays were conducted to explore the migration and invasion ability after co-transfected with pcDNA3.1-KTN1-AS1 and si-RBBP4 in Kyse150 cells. Error bars are shown as mean ± SD from three replicate experiments (n = 3) (*P < 0.05, **P < 0.01, ***P < 0.001).

For the molecular mechanism by which *KTN1-AS1* affects the expression of *E-cadherin*, we further verified in terms of cellular phenotype. The overexpression plasmid of *KTN1-AS1* was co-transfected with si-RBBP4 into Kyse150 and Kyse170 cells to investigate their effects on cell function. Reduced *RBBP4* expression partially reversed the enhanced proliferation, migration, and invasion ability induced by overexpression of *KTN1-AS1* (Fig. 6A–D and Supplementary Fig. S4A–D), indicating that the effect of *KTN1-AS1* on the malignant behavior of esophageal cancer cells is affected by RBBP4.

### *KTN1-AS1* promotes ESCC cell growth in vivo

**Figure 7:**
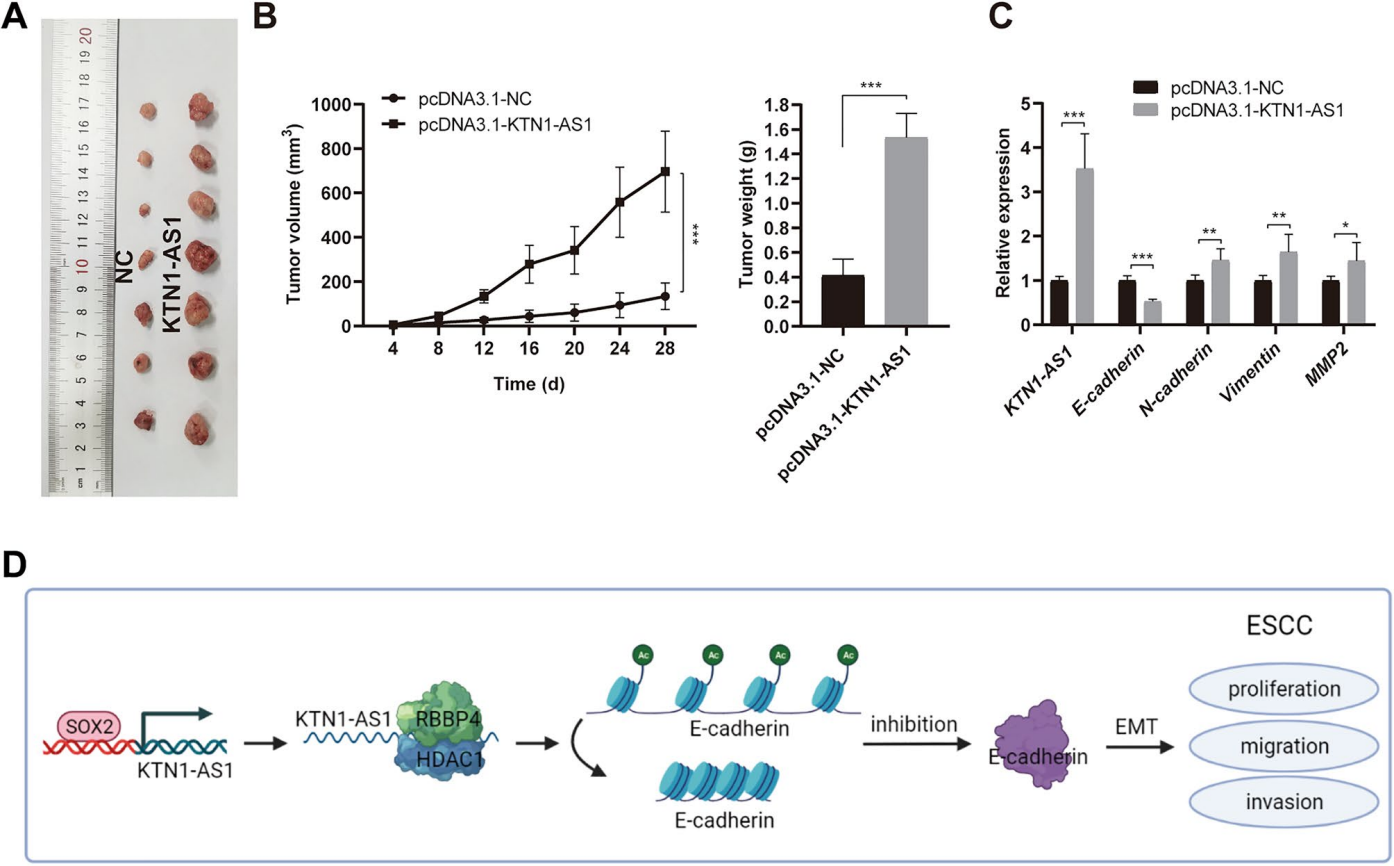
*KTN1-AS1* promotes esophageal squamous cell carcinoma (ESCC) cell growth in vivo. (**A**) Xenograft tumor images dissected from nude mice after stable overexpression of *KTN1-AS1*. (**B**) The tumor volume and weight of different groups (n = 7). (**C**) The relative expression of *KTN1-AS1*, *E-cadherin*, *N-cadherin*, *Vimentin*, and *MMP2* in xenograft tissues detected by qRT-PCR method. (**D**) The possible mechanism of *KTN1-AS1* regulates ESCC progression. Error bars are shown as mean ± SD from three replicate experiments (n = 3) (*P < 0.05, **P < 0.01, ***P < 0.001).

To further verify the carcinogenic effect of *KTN1-AS1* on ESCC, the tumor xenograft experiments were conducted to investigate the effects of *KTN1-AS1* on tumor growth in vivo. Compared with control group, the tumor size, tumor volume and weight in the *KTN1-AS1* upregulated group were significantly increased (Fig. 7A,B). Furthermore, the expression of *KTN1-AS1* in the xenograft tissues of the *KTN1-AS1* overexpression group was increased, accompanied by the increased mRNA expression level of *N-cadherin*, *Vimentin*, and *MMP2*, while the expression of *E-cadherin* was decreased (Fig. 7C). All these findings indicated that *KTN1-AS1* could promote ESCC tumor growth in vivo.

In conclusion, all these findings suggested that *KTN1-AS1* might promote ESCC progression by activating EMT process via RBBP4/HDAC1/E-cadherin axis (Fig. 7D).

## Discussion

It is well known that lncRNAs take up a significant proportion acting as either tumor suppressors or oncogenes in the tumor carcinogenesis. In this study, *KTN1-AS1* was proved to be upregulated in esophageal cancer tissues and cells, and exhibited as an oncogene in facilitating ESCC cells proliferation, migration, and invasion. The expression of *KTN1-AS1* maybe induced by SOX2, and has a relationship with the EMT process.

In non-small cell lung cancer (NSCLC), signal transducer and activator of transcription 1 (STAT1) was proved to bind to the promoter region of *KTN1-AS1* and activated its transcription^11^. Since the promoter region of *KTN1-AS1* is riched in transcriptional regulatory elements, we speculated that there should be other transcriptional factors involved in the regulation of abnormal *KTN1-AS1* expression in ESCC. Amplification of SOX2 is one of the gene characteristics of ESCC and the expression of SOX2 is specifically highly in ESCC^21^. In the present study SOX2 was proved to activate transcription of *KTN1-AS1.* In addition, SOX2 acts as a potential EMT-inducing transcriptional factor in promoting cancer cells invasion and metastasis, and SOX2 induced lncRNAs have been demonstrated to play pivotal roles in tumorigenesis^22–24^, so there may be potential promoting role for *KTN1-AS1* on ESCC cells progression.

Nuclear-localized lncRNAs can recruit chromatin modification and remodeling complexes to specific genomic sites to change the chromosome structure and modification status, and DNA/RNA methylation status, and further control the related genes expression^25^. Accumulating evidence has demonstrated that lncRNAs participate in these processes by binding to specific proteins^26^. Our present study found that *KTN1-AS1* could bind with RBBP4 in the nucleus. As a chaperone protein, RBBP4 exerts its oncogenic function by participating in the formation of gene regulatory complexes, such as polycomb repressive complex 2 (PRC2)^27^, nucleosome remodeling factor (NURF)^28^, NuRD^29^, Sin3 complex^19^, and the deacetylase module (HDAC1/2, MTA1/2/3, RBBP4/7) complex^30,31^. Previous studies of *KTN1-AS1* have primarily focused on the mechanism of competing endogenous RNA (ceRNA). In this study, we first discussed the underlying mechanism between *KTN1-AS1* and RNA-binding protein in ESCC.

EMT is a cellular program known to be critical for malignant progression of tumors^32^. Since *KTN1-AS1* could regulate the expression of EMT-related genes, we paid more attention to whether *KTN1-AS1* regulates the expression of EMT-related genes through RBBP4 and HDAC1. E-cadherin, a typical epithelial cell marker, is a Ca^2+^ dependent transmembrane glycoprotein closely related to intercellular adhesion. The dysregulation of E-cadherin expression that leads to carcinogenesis happens mostly at the epigenetic level^33,34^. HDACs are enzymes mediating the removal of acetyl from lysine residues in either histones or other proteins, causing the repression of gene transcription and subsequent changes in signaling events^35^, and HDAC1 was demonstrated to be involved in the transcriptional regulation of *E-cadherin* expression^17,18^. Our subsequent studies found that *KTN1-AS1* could also bind to HDAC1 and affect the binding ability of RBBP4 to HDAC1. *KTN1-AS1* may silence the expression of *E-cadherin* by forming a complex with RBBP4 and HDAC1 and enhancing its deacetylation effect on the promoter of *E-cadherin* in the nucleus, thereby promoting the EMT process in ESCC.

## Conclusions

In summary, this study identifies the novel oncogenic role of lncRNA *KTN1-AS1* in ESCC. Transcriptionally activated *KTN1-AS1* by SOX2 may silence the expression of *E-cadherin* at epigenetic level by binding with RBBP4 and HDAC1 in the nucleus. *KTN1-AS1* may act as a potential therapeutic and prognostic biomarker for ESCC.

## Methods

### Clinical specimens

One hundred and eleven ESCC patients were included in this study. The patients didn’t undergo any radiotherapy or chemotherapy before operation between the years of 2013 to 2015 in the Fourth Hospital of Hebei Medical University. The clinical information was collected from the hospital records. The study was reported in accordance with ARRIVE guidelines and informed consent was obtained from all patients. This study was approved by the Ethics Committee of the Fourth Hospital of Hebei Medical University.

### Cell culture and treatment

Human esophageal cancer cell lines (Kyse150, Kyse170, Eca109, and TE1) were purchased from American Type Culture Collection (ATCC). Cells were cultured in RPMI-1640 (Invitrogen, Carlsbad, CA, USA) containing 10% heat-inactivated fetal bovine serum (Invitrogen) in an atmosphere containing 5% CO_2_ at 37 °C. For Trichostatin A (TSA) (CAS No. 58880-19-6, Sigma) treatment, Kyse 150 and Kyse170 cells were transfected for 12–24 h, then treated with 300 nM TSA for additional 48 h.

### RNA extraction and quantitative real-time polymerase chain reaction (qRT-PCR) assay

The TRIzol reagent (Solarbio, Beijing, China) was used to extract RNA from tissues and cells. The cDNA was synthesized using the Transcriptor First Strand cDNA Synthesis Kit (Roche, Basel, Switzerland). GoTap^®^qPCR Master Mix (Promega, Madison, WI, USA) was used to perform the quantitative real time PCR in the StepOne plus Real-Time PCR System (Applied Biosystems). The relative expression levels of mRNA and lncRNA were normalized to *GAPDH* as endogenous control respectively by using the 2^−△△Ct^ method. Primer sequences were listed in Supplementary Table S1.

### Cell transfection

The pcDNA3.1-KTN1-AS1 was purchased from GenScript (Nanjing, China). For overexpression of *SOX2*, the cDNA encoding *SOX2* was amplified and was inserted into pcDNA3.1 vector (Invitrogen), named pcDNA3.1-SOX2. The SOX2 siRNA, KTN1-AS1 siRNA, RBBP4 siRNA, and si-NC were purchased from GenePharma (Shanghai, China), and the sequences were listed in Supplementary Table S2. Lipofectamine 2000 (Invitrogen) was used to perform the transfection. The transfection efficiency was detected by qRT-PCR method.

### Cell proliferation assay

The cellular proliferation capability was detected using MTS and clone formation assays. For MTS assay, 1 × 10^3^ cells after 24 h of transfection were seeded into 96-well plate. The CellTiter 96^®^AQ_ueous_ One Solution Reagent (Promega) was added after incubation of 0 h, 24 h, 48 h, 72 h, and 96 h. Then the optical density for each well was measured after incubation for 2 h. As to clone formation assay, 3 × 10^3^/5 × 10^3^ cells after 24 h of transfection were seeded into a 6-well plate and were routine cultured for 1 week. The 4% paraformaldehyde was used to fix the cells and stained with crystal violet solution.

### Cell migration and invasion assay

Wound healing and transwell assays were performed to detect the migration and invasion ability. For wound healing assay, transfected cells were inoculated in a 6-well plate. A straight scratch was made in each well when the cell density was close to overgrown, and pictures were taken at the same position at 0 h, 12 h, and 24 h using a microscope. For transwell assay, 1 × 10^5^ cells after 24 h of transfection were seeded onto the upper compartment of the matrigel-coated chamber (Corning Costar, Corning, NY, USA) with 200 μL of serum-free RPMI-1640 medium; in the lower chamber, 600 μL of the medium containing 10% fetal bovine serum were added. Cells on the upper surface were wiped off after 24 h of incubation; the 4% paraformaldehyde was used to fix the invasive cells on the lower surface of the chamber and stained with crystal violet solution.

### Western blot assay

The RIPA lysis buffer and PMSF (Solarbio) were used to extract proteins from cells. BCA Protein Assay Kit (Multi Sciences, Hangzhou, China) was used to detect the protein concentration. The protein lysates were transferred to PVDF membranes (Millipore, Sigma, Burlington, MA, USA) after separated by 10% polyacrylamide gel electrophoresis. The enhanced chemiluminescence detection reagent (Multi Sciences) was used to detect the protein bands by Chemi XT 4 (Syngene). The main antibodies were listed as follows: anti-β-actin (ZenBioScience, Cat# 380624), anti-E-cadherin (ZenBioScience, Cat# R22490), anti-N-cadherin (ZenBioScience, Cat# 380671), anti-Vimentin (ZenBioScience, Cat# R22775), anti-MMP2 (ZenBioScience, Cat# 380817), anti-RBBP4 (ZenBioScience, Cat# 385565), anti-HDAC1 (Proteintech, Cat# 10197-1-AP).

### Subcellular fractionation assay

The nuclear and cytoplasm fractions of esophageal cancer cell lines were isolated by PARIS™ Kit Protein and RNA Isolation System (Invitrogen). The subcellular localization of *KTN1-AS1* was detected by qRT-PCR method. *GAPDH* and *U6* were used as control genes expressed in cytoplasm and nucleus, respectively.

### Vector construction

The fragments of *KTN1-AS1* containing the predicted binding sites of SOX2 were amplified and inserted into the pGL3-basic vector (Promega). The point mutations of the binding sites were performed using Q5^®^ Site-Directed Mutagenesis Kit (New England Biolabs). Mutation primers were designed in NEBase changer (NEBaseChanger). Primers used for fragment amplification were listed in Supplementary Table S3. All recombinant plasmids were sequenced correctly.

### RNA pull-down assay

Full-length sense and antisense *KTN1-AS1* sequences were obtained using RiboMAX™ Large Scale RNA Production System-T7 (Promega). The Pierce™ RNA 3′ End Desthiobiotinylation Kit (Thermo Fisher Scientific) was then applied to biotin-label the obtained RNA sequence. The purified biotin-labeled RNA was incubated with magnetic beads and protein lysate using the Pierce™ Magnetic RNA–Protein Pull-Down Kit (Thermo Fisher Scientific). Proteins were separated using polyacrylamide gel electrophoresis and stained with Coomassie brilliant blue (Beyotime, Jiangsu, China). The different bands between sense and antisense *KTN1-AS1* were verified using mass spectrometry and detected through western blot analysis.

### Luciferase reporter assay

In Kyse150 and Kyse170 cells, the promoter-reporter gene plasmids were separately co-transfected with pcDNA3.1-SOX2 or pcDNA3.1 empty plasmid. After 48 h of transfection, the luciferase activity was detected using the Dual-Luciferase Reporter Assay System (Promega) and normalized to Renilla activity.

### Chromatin immunoprecipitation (ChIP) assay

ChIP assay was carried out using the EZ-ChIP™ kit (Millipore). After sonicating the cross-linked chromatin DNA, the antibodies against SOX2 (ZenBioScience, Cat# 864316) and acetyl histones H3 (Active motif, Ca# 61937) were used for precipitating the DNA fragments overnight at 4 °C, and protein A/G agarose beads were added to collect the precipitated complexes. The precipitated DNA fragment was purified and subjected to PCR detection. Primers used for the ChIP sequence were listed in Supplementary Table S4.

### RNA immunoprecipitation (RIP) assay

Cells were re-suspended in NP-40 lysis buffer. RNA was immunoprecipitated with antibodies against RBBP4 (ZenBioScience, Cat# 385565) and HDAC1 (Proteintech, Cat# 10197-1-AP). The qRT-PCR method was performed to detect the expression of *KTN1-AS1*.

### Co-immunoprecipitation (Co-IP) assay

Cells were lysed with NP-40 lysis buffer and the lysates were precleared with protein A/G agarose beads (Santa Cruz Biotechnology, Dallas, Texas, USA). The supernatant was incubated with an antibody against RBBP4 overnight at 4 ℃ and protein A/G agarose beads were then added for further incubation. The precipitates were washed with lysis buffer and then suspended in 5 × SDS-PAGE sample loading buffer. After boiling for 10 min, the samples were analyzed by western blot and detected by the relevant antibodies.

### Tumor xenograft model

G-418 bioreagent (Merck, Rahway, NJ, USA) was used to generate *KTN1-AS1* stable expression Kyse150 cells. A total of 5 × 10^6^ cells were subcutaneously injected into one side of male BALB/c-nude mice purchased from Beijing HFK Bioscience CO., Ltd. Tumor volume was measured and calculated every 4 days. The mice were dissected on the 28 days, and tumors weight were measured. The animal experiments were conducted at the Experimental Animal Center of the Fourth Hospital of Hebei Medical University under the guidelines of NIH, and this study was approved by the Committee on the Ethics of Animal Experiments of the Fourth Hospital of Hebei Medical University.

### Statistical analysis

SPSS 22.0 software package and GraphPad Prism 8.0 were used to perform data analysis and graphing. The statistical differences analysis between the two groups was conducted using Student's t-test. For overall survival analysis, Kaplan–Meier method and log-rank test were used. Univariate and multivariate Cox regression analysis was used to investigate the independent prognostic parameters. All experiments data came from three independent experiments performed in duplicate and presented as mean ± SD. *P* < 0.05 was considered statistically significant.

### Ethics statement

The study involving the usage of patients tissues was performed in accordance with the Declaration of Helsinki and was approved by the Ethics Committee of the Fourth Hospital of Hebei Medical University. The study was reported in accordance with ARRIVE guidelines.

## Supplementary Information

Supplementary Figures.

Supplementary Tables.

## Data Availability

The datasets analyzed during the current study are available from the corresponding author on reasonable request.
