# Supplementary material for: KTN1-AS1, a SOX2-mediated lncRNA, activates epithelial–mesenchymal transition process in esophageal squamous cell carcinoma
Source: Sci Rep. 2022 Nov 23;12:20186. doi: 10.1038/s41598-022-24743-z (PMC9684558; doi:10.1038/s41598-022-24743-z)
Supplement: Supplementary file 1 — Supplementary Figures. [file 41598_2022_24743_MOESM1_ESM.docx]

**Supplementary Figures**

**A**


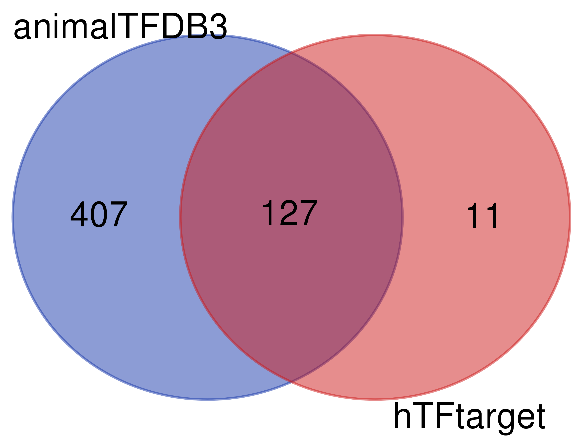


**B**

| R＞0.3 | CTCF MAZ NOTCH1 SMAD3 GTF3C2 MYC CEBPB SMAD2 BCL6 UBTF SREBF SIN3A E2F4 RAD21 BRD4 SOX2 SMC3 YY1 POLR2A TFAP2A RUNX1 HIF1A TFAP2C REST SMAD1 FOXM1 MXI1 TFAP4 SIX5 SUMO2 RUNX3 CDK9 MAX CBFB RCOR1 KDM5B |
| --- | --- |

**Supplementary Figure S1 Potential transcription factors of *KTN1-AS1* predicted by animalTFDB3 and hTFtarget.** (A) Venn diagram made by the potential transcription factors of *KTN1-AS1* predicted by animalTFDB3 and hTFtarget. (B) Transcription factors with an R value greater than 0.3 among the 127 transcription factors predicted by animalTFDB3 and hTFtarget.


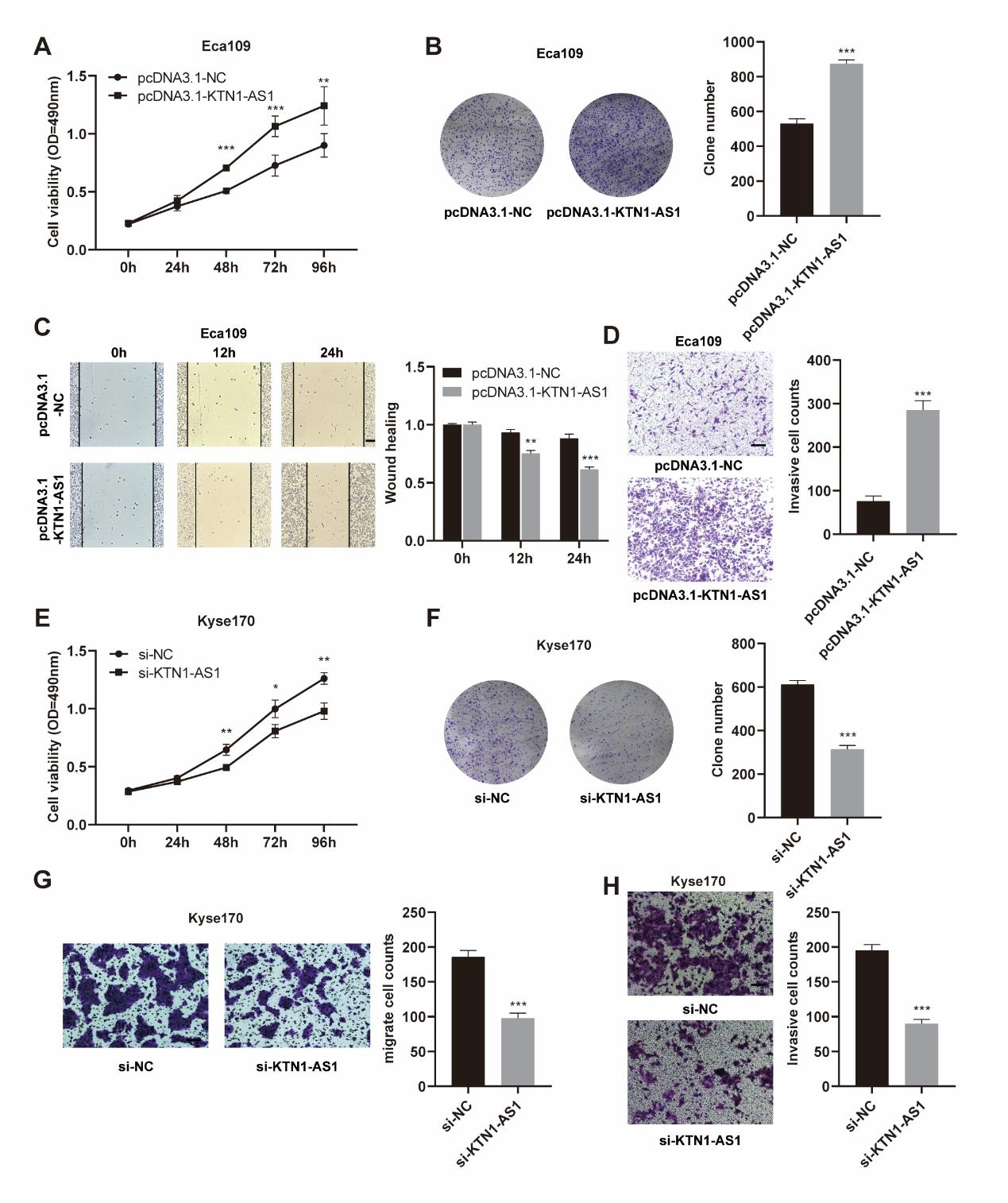
**Supplementary Figure S2. *KTN1-AS1* facilitates ESCC cells proliferation, migration, and invasion.** (A, B) MTS and clone formation assays demonstrate the cell proliferation ability in Eca109 cells with upregulation of *KTN1-AS1*. (C, D) Wound healing and transwell invasion assays indicate the enhanced migration and invasion ability of Eca109 cells with upregulated *KTN1-AS1*. (E, F) MTS and clone formation assays demonstrate the cell proliferation ability in Kyse170 cells with downregulation of *KTN1-AS1*. (G, H) Transwell migration and invasion assays indicate the decreased migration and invasion ability in Kyse170 cells with downregulated *KTN1-AS1*. Error bars are shown as mean ± SD from three replicate experiments (n = 3) (**P* < 0.05, ***P* < 0.01, ****P* < 0.001).


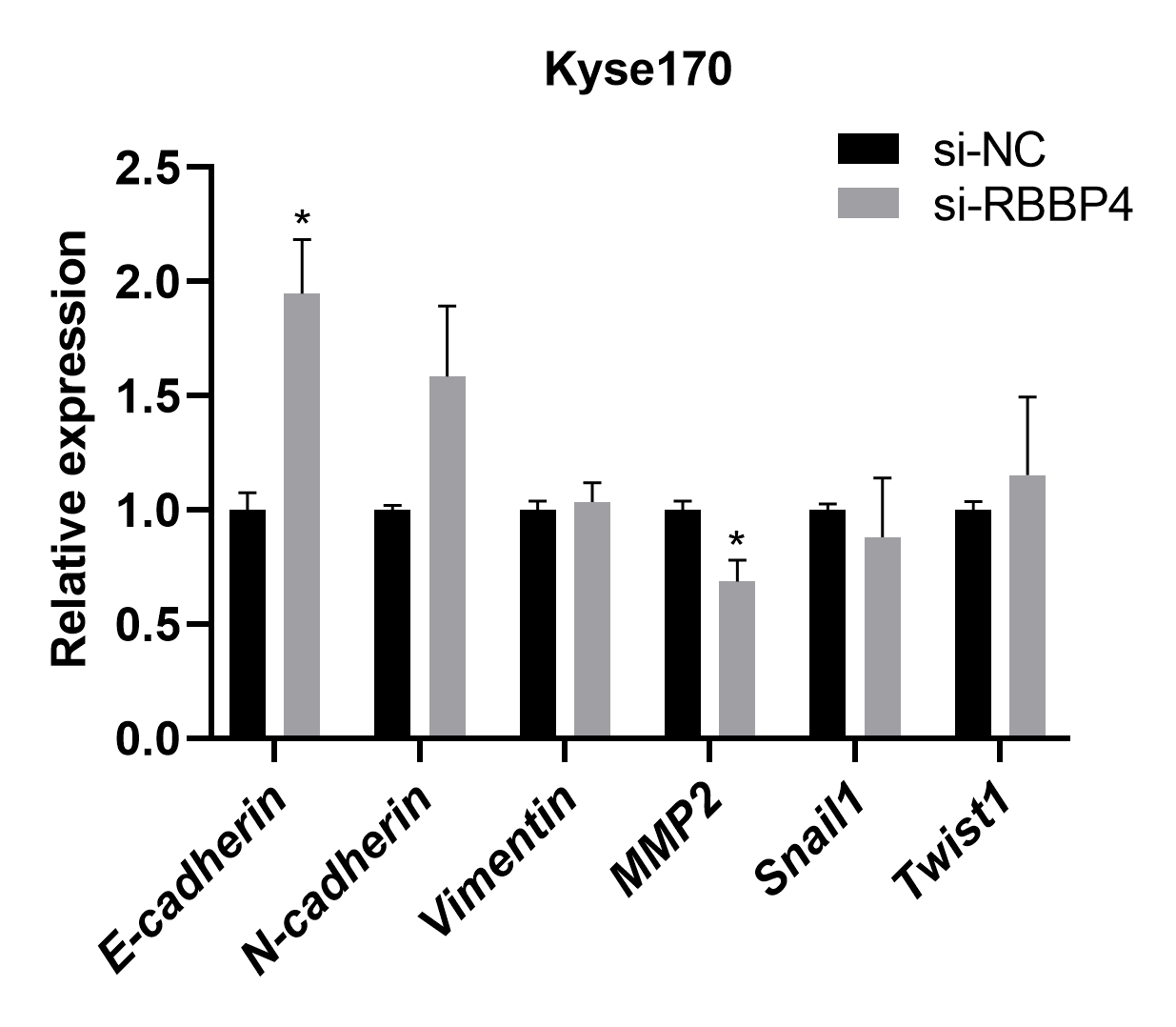
**Supplementary Figure S3** The effect of si-RBBP4 on some EMT related markers, such as *E-cadherin*, *N-cadherin*, *Vimentin*, *MMP2*, *Snail1*, and *Twist1*. (**P* < 0.05).


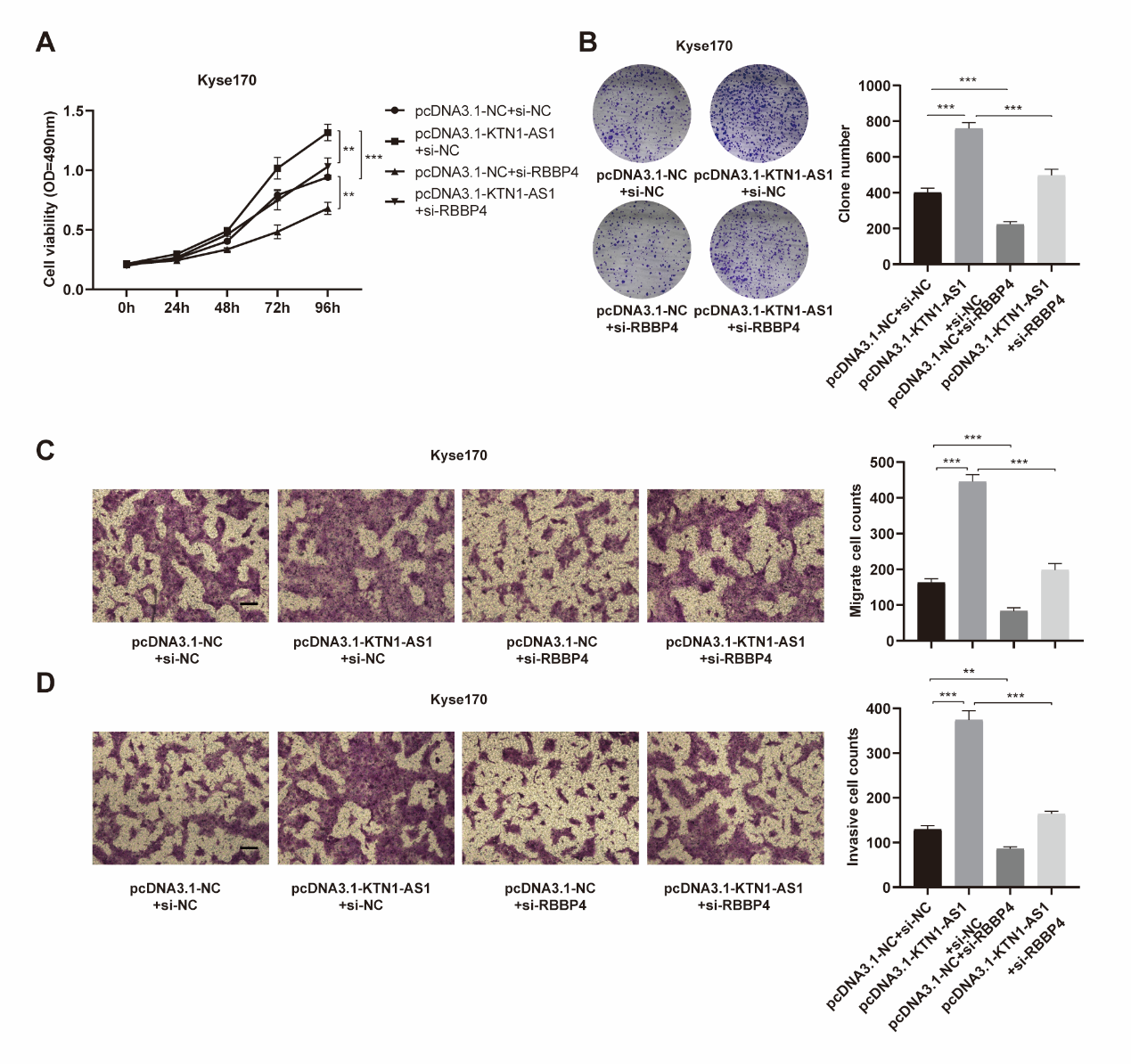
**Supplementary Figure S4. RBBP4 partially reverses the biological function of *KTN1-AS1* on esophageal squamous cell carcinoma (ESCC) cells.** (A, B) MTS and clone formation assays were performed to analyze the cell proliferation ability after co-transfected with pcDNA3.1-KTN1-AS1 and si-RBBP4 in Kyse170 cells. (C, D) Transwell migration and invasion assays were conducted to explore the migration and invasion ability after co-transfected with pcDNA3.1-KTN1-AS1 and si-RBBP4 in Kyse170 cells. Error bars are shown as mean ± SD from three replicate experiments (n = 3) (**P* < 0.05, ***P* < 0.01, ****P* < 0.001).

**A**

**
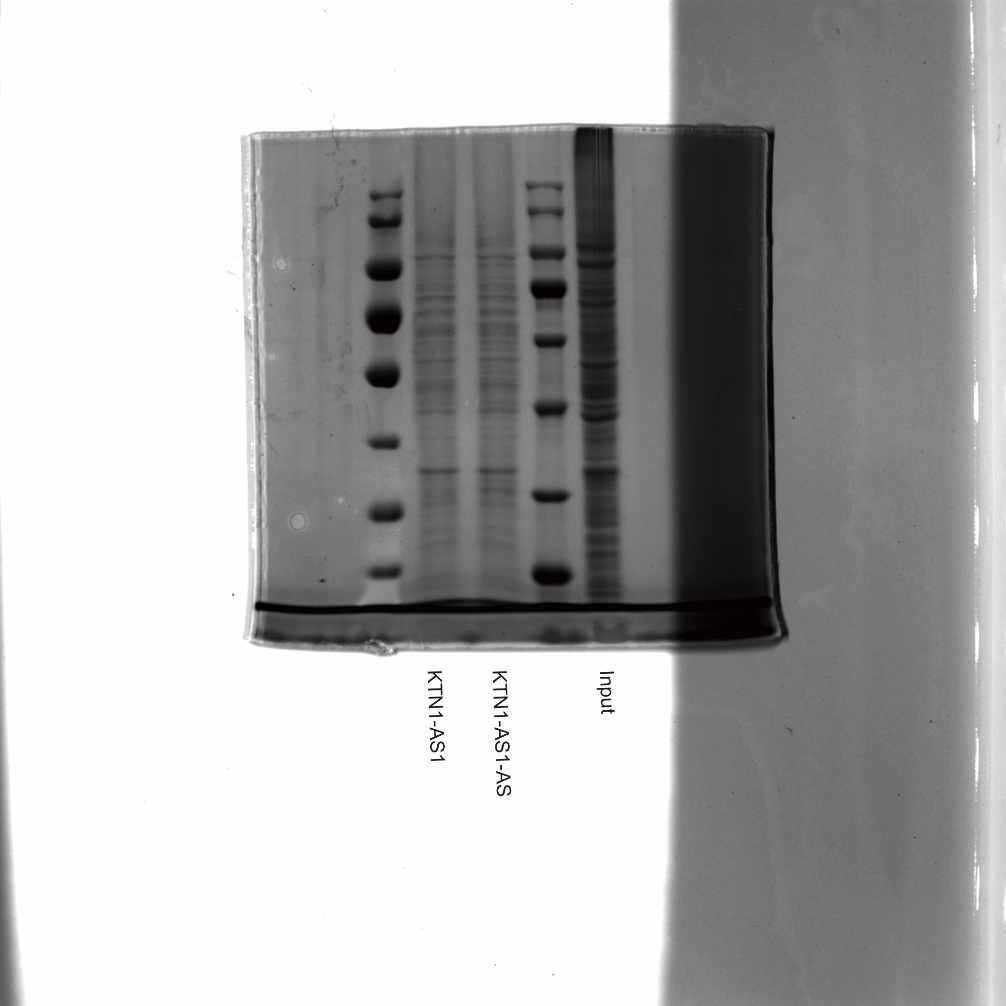
**

Original protein bands in Fig. 4B

**B**


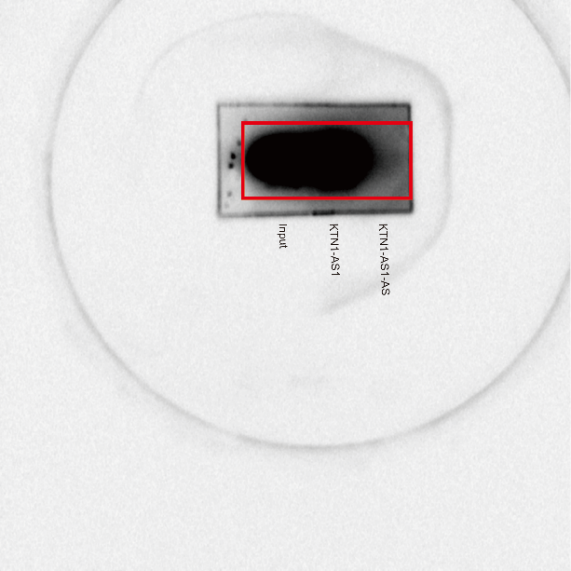


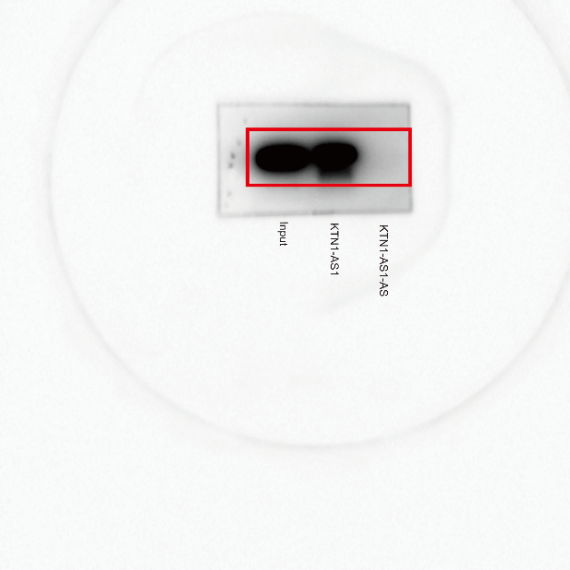


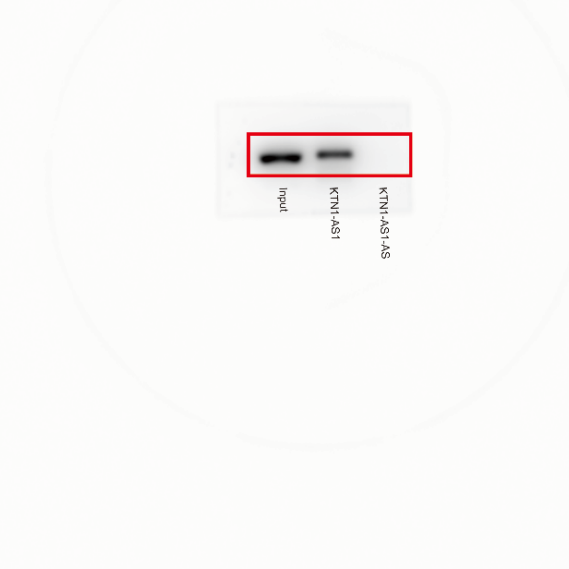


RBBP4 (protein bands of RBBP4 under different exposure intensities in Kyse150 group in Fig. 4C)


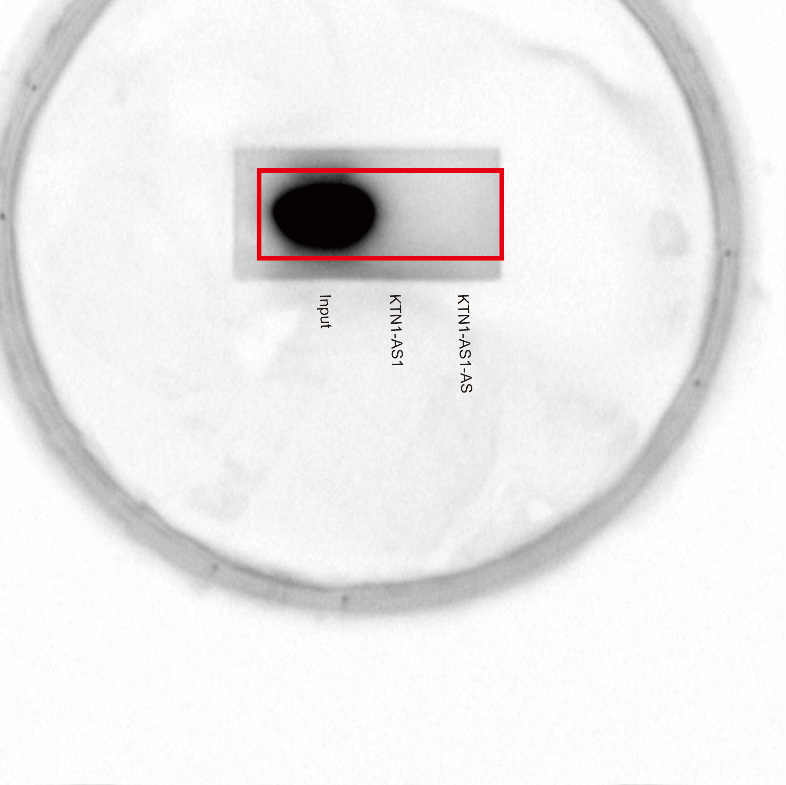


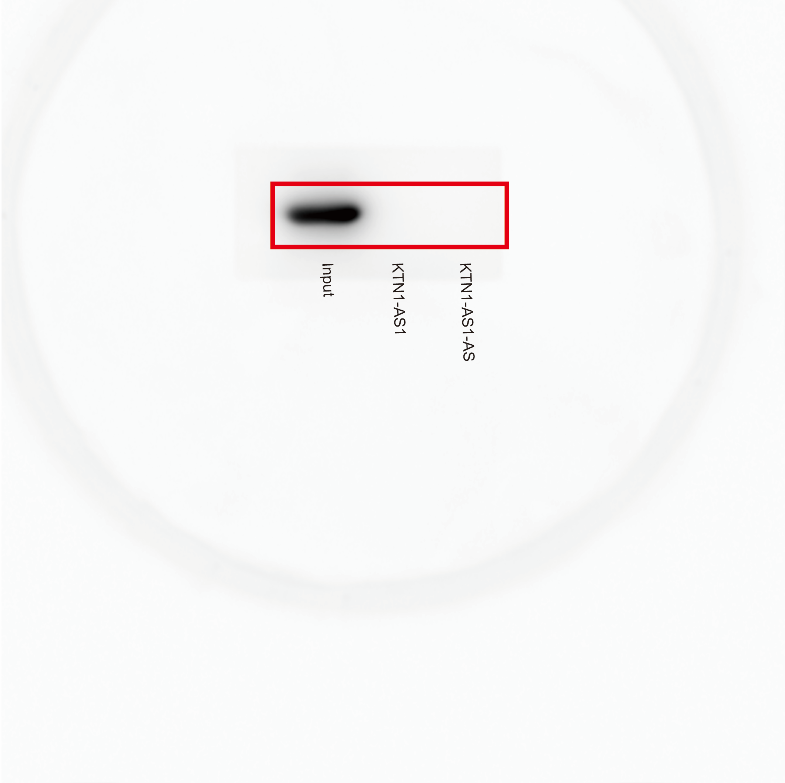


ACTB (protein bands of ACTB under different exposure intensities in Kyse150 group in Fig. 4C)


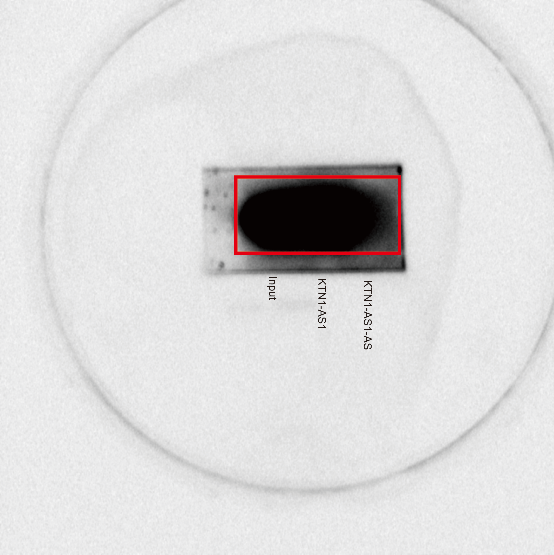


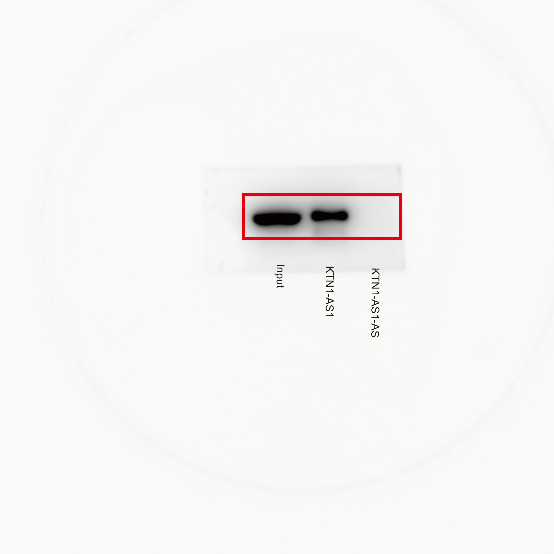


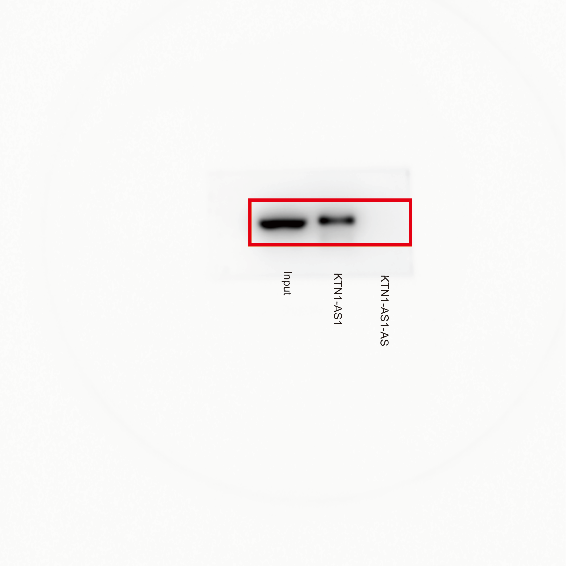


RBBP4 (protein bands of RBBP4 under different exposure intensities in Kyse170 group in Fig. 4C)


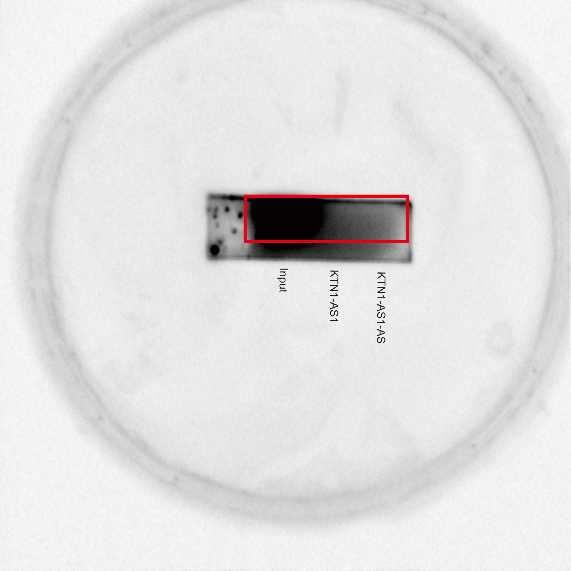


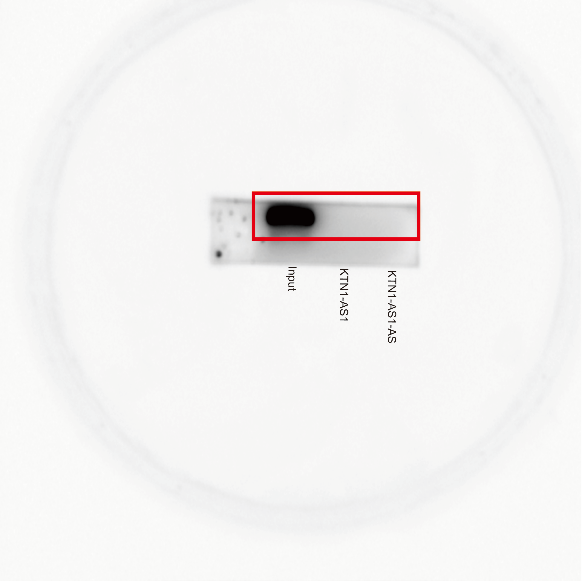


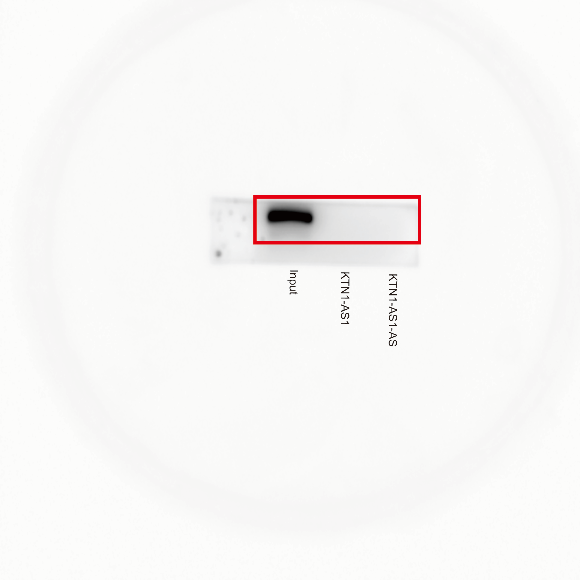


ACTB (protein bands of ACTB under different exposure intensities in Kyse170 group in Fig. 4C)

**C**


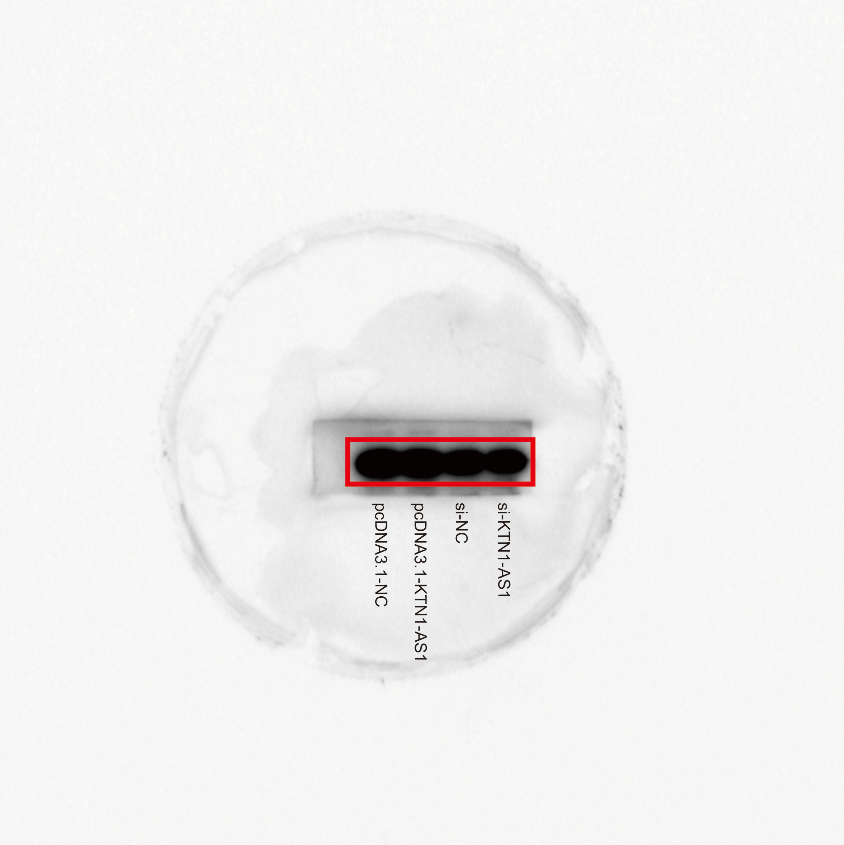


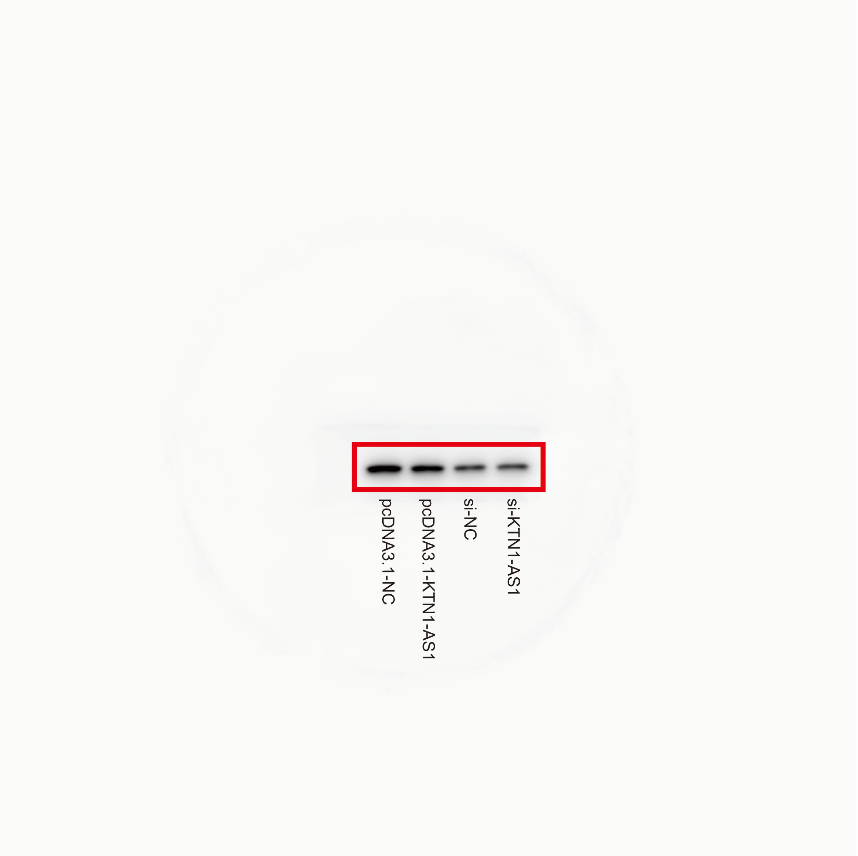


RBBP4 (protein bands of RBBP4 under different exposure intensities in Fig. 4E)


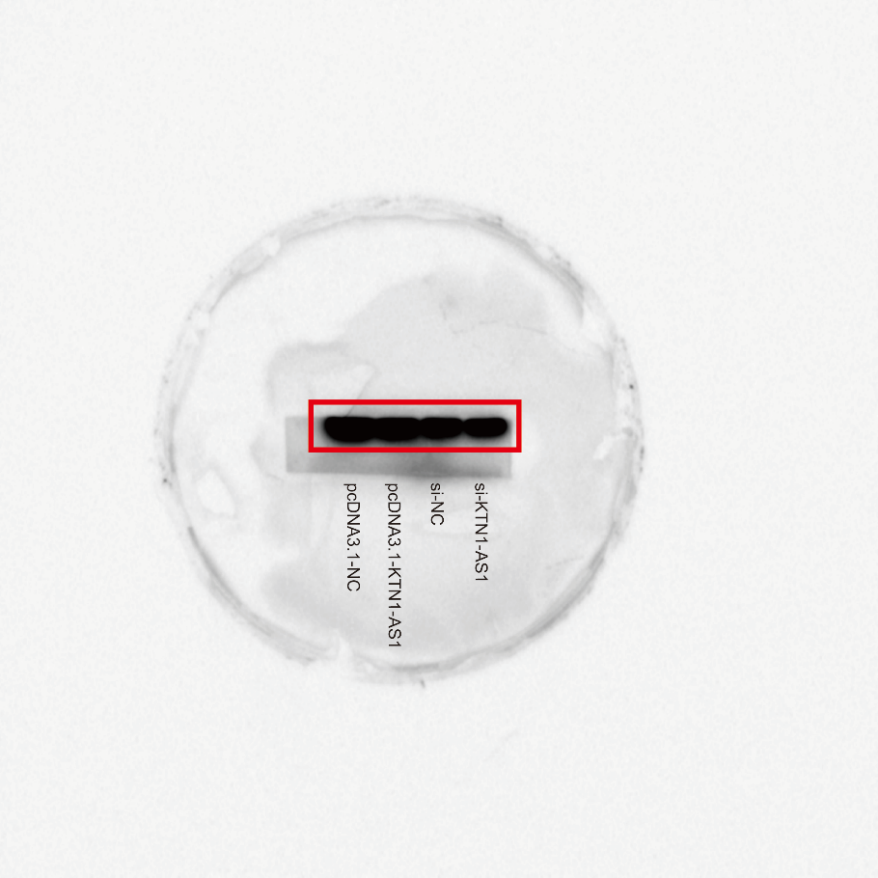


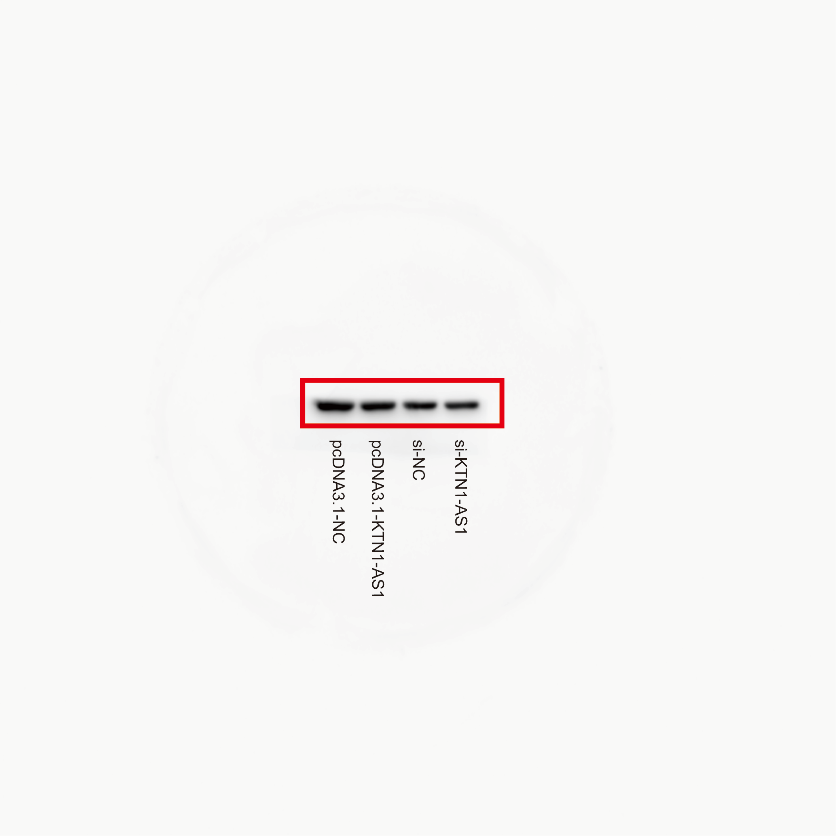


ACTB (protein bands of ACTB under different exposure intensities in Fig. 4E)

**D**


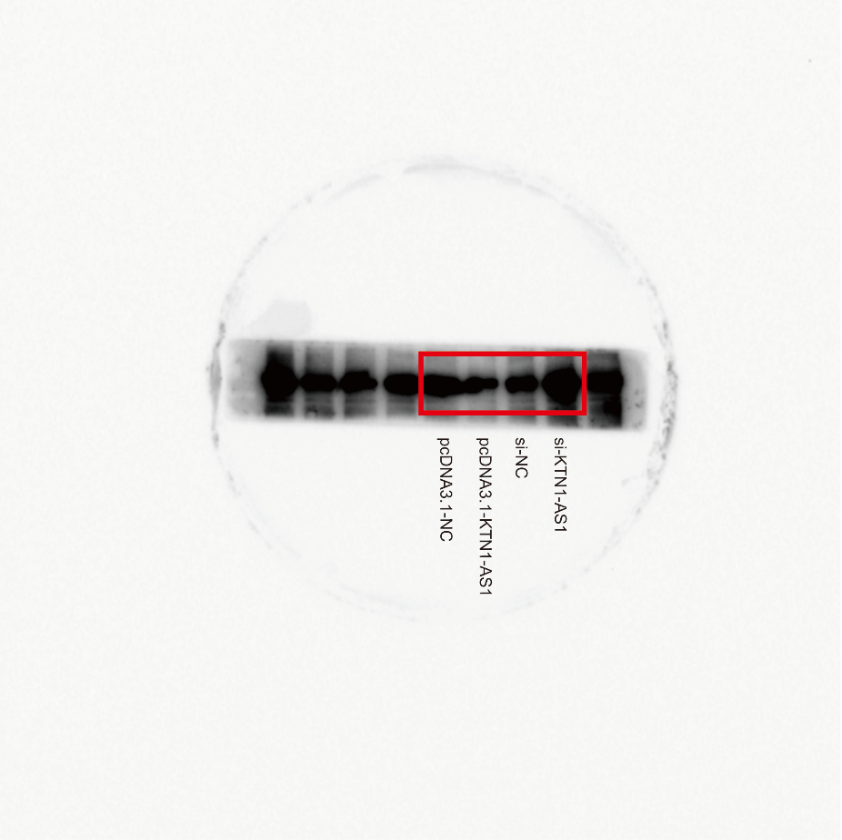


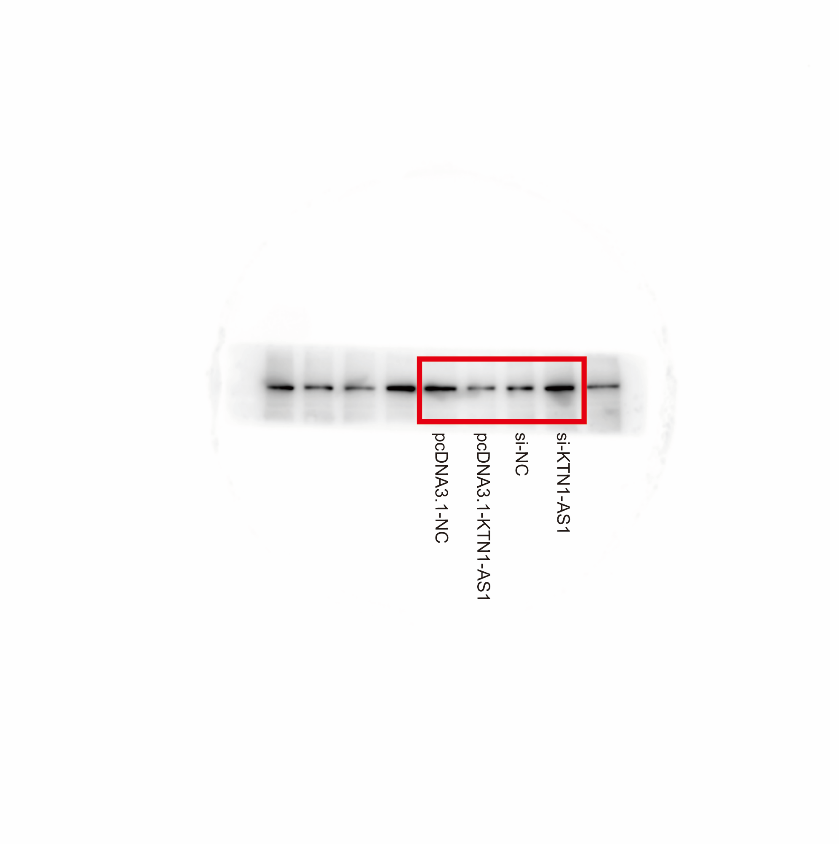


E-cadherin (protein bands of E-cadherin under different exposure intensities in Fig. 5B)


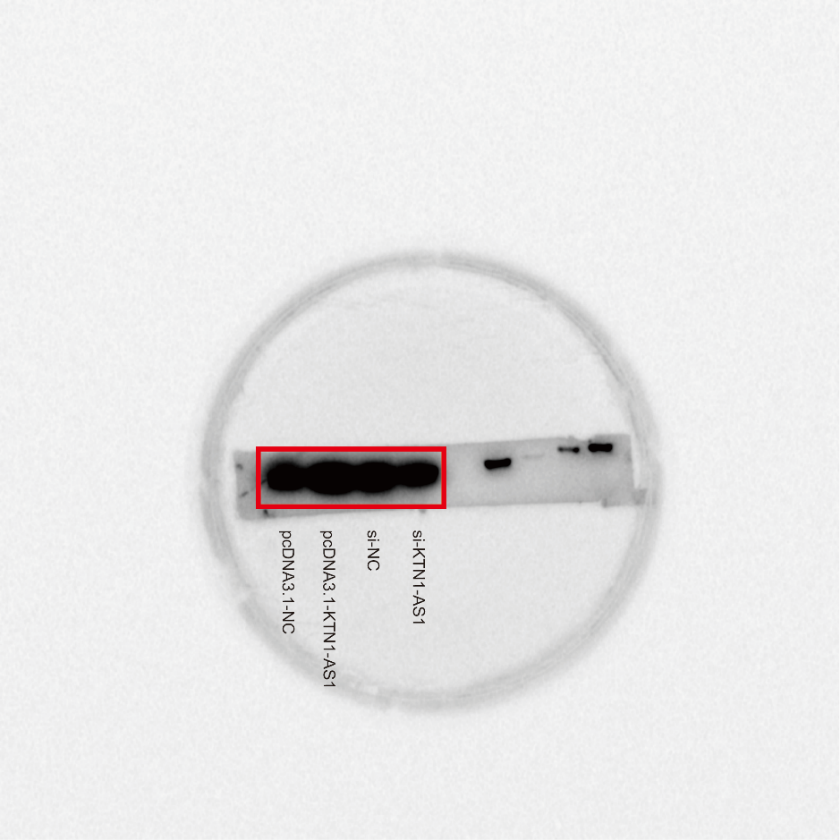


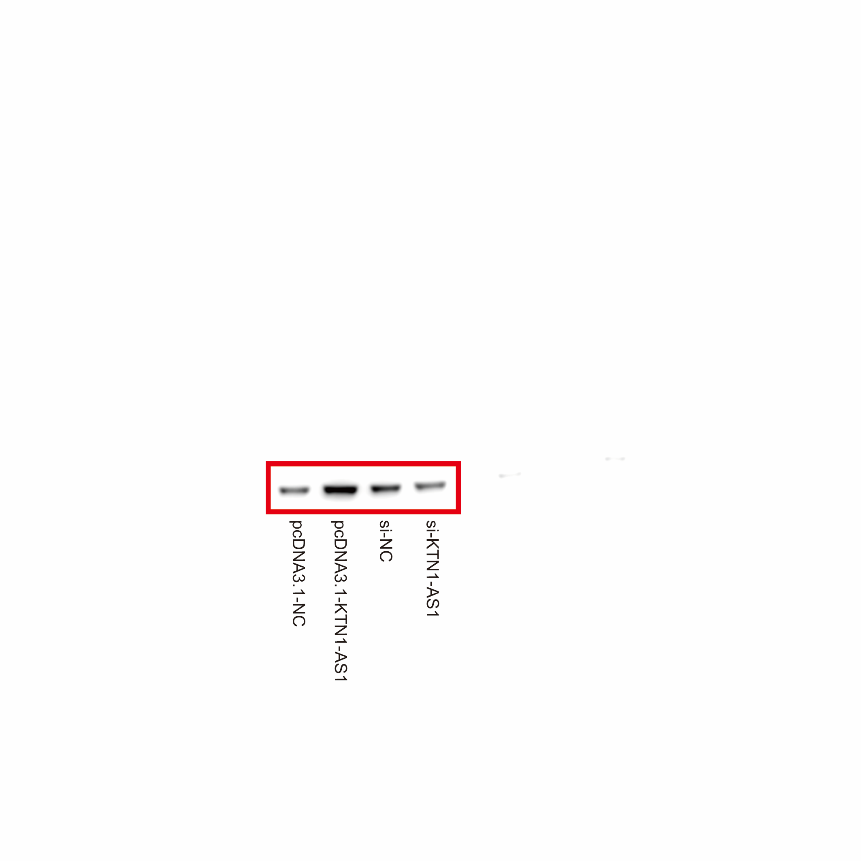


N-cadherin (protein bands of N-cadherin under different exposure intensities in Fig. 5B)


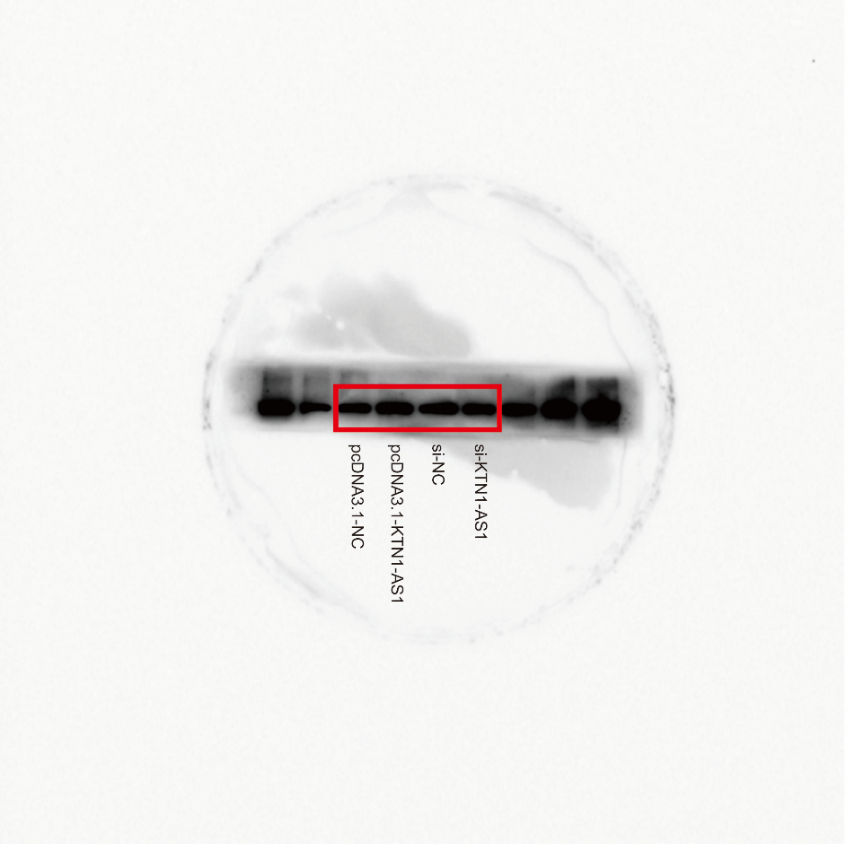


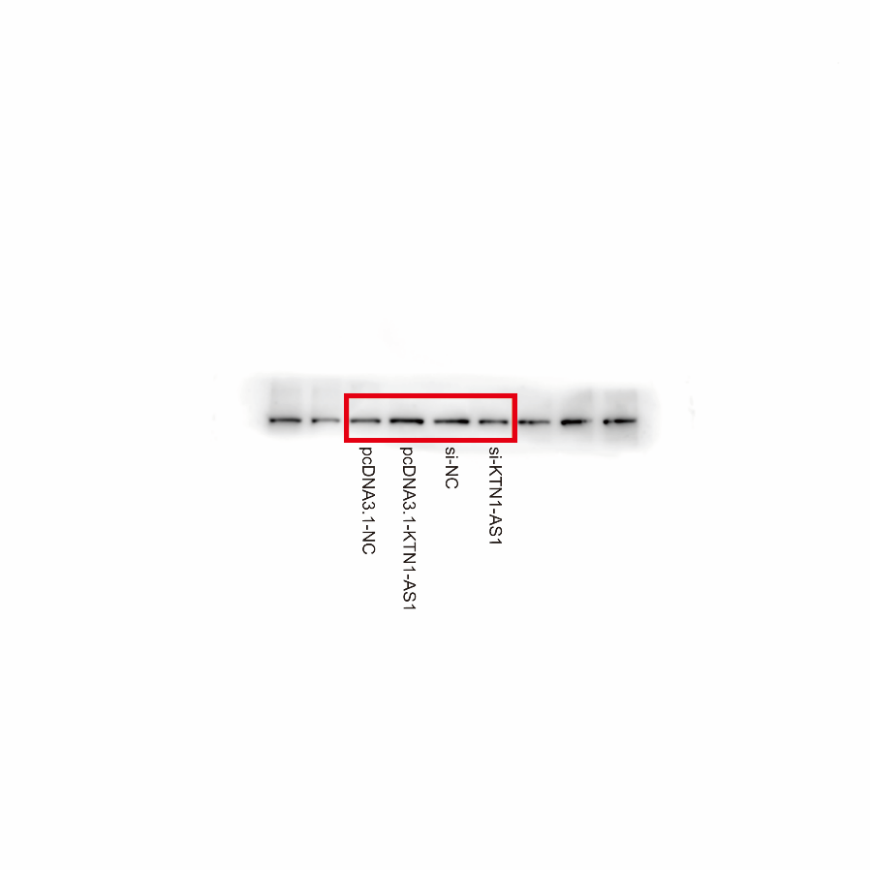


Vimentin (protein bands of Vimentin under different exposure intensities in Fig. 5B)


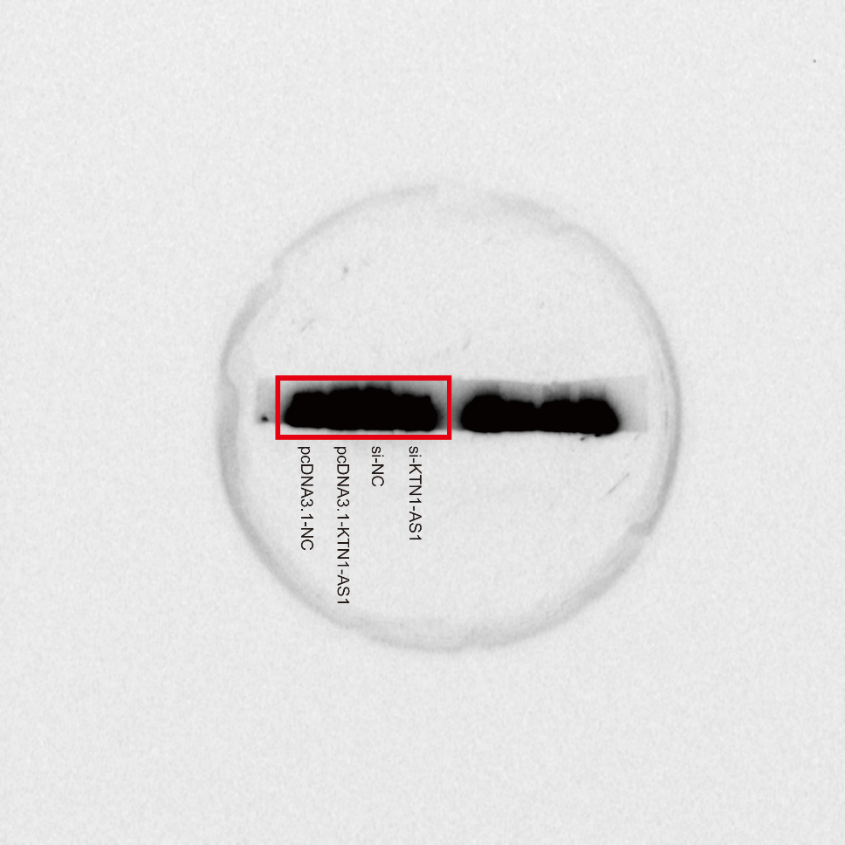


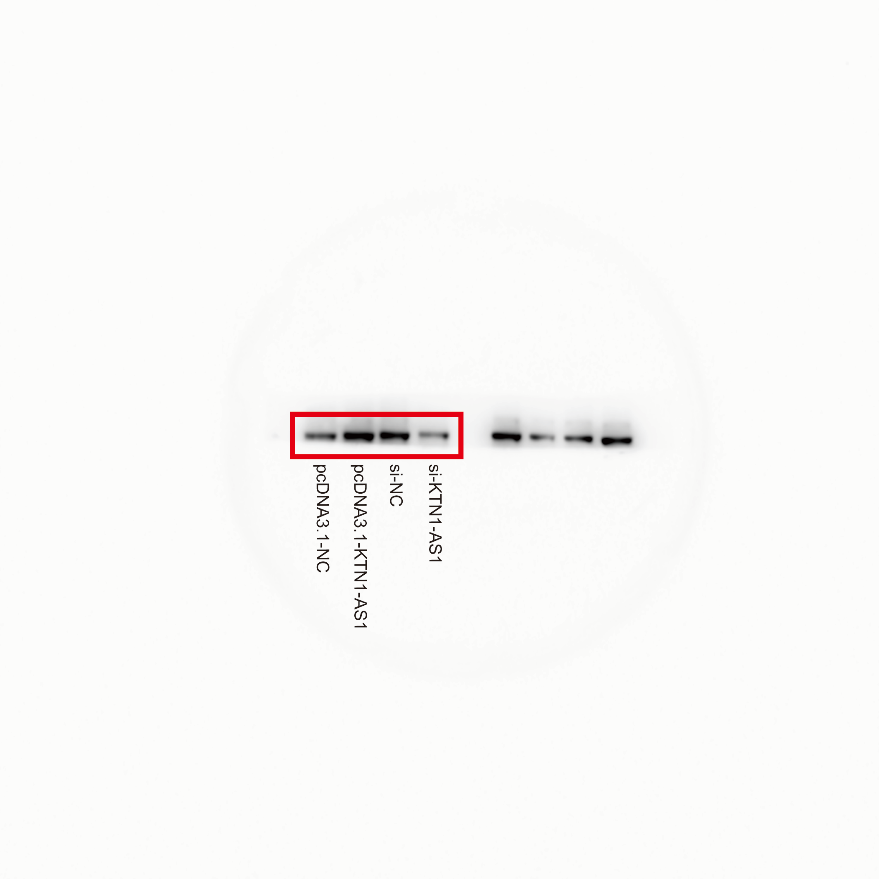


MMP2 (protein bands of MMP2 under different exposure intensities in Fig. 5B)


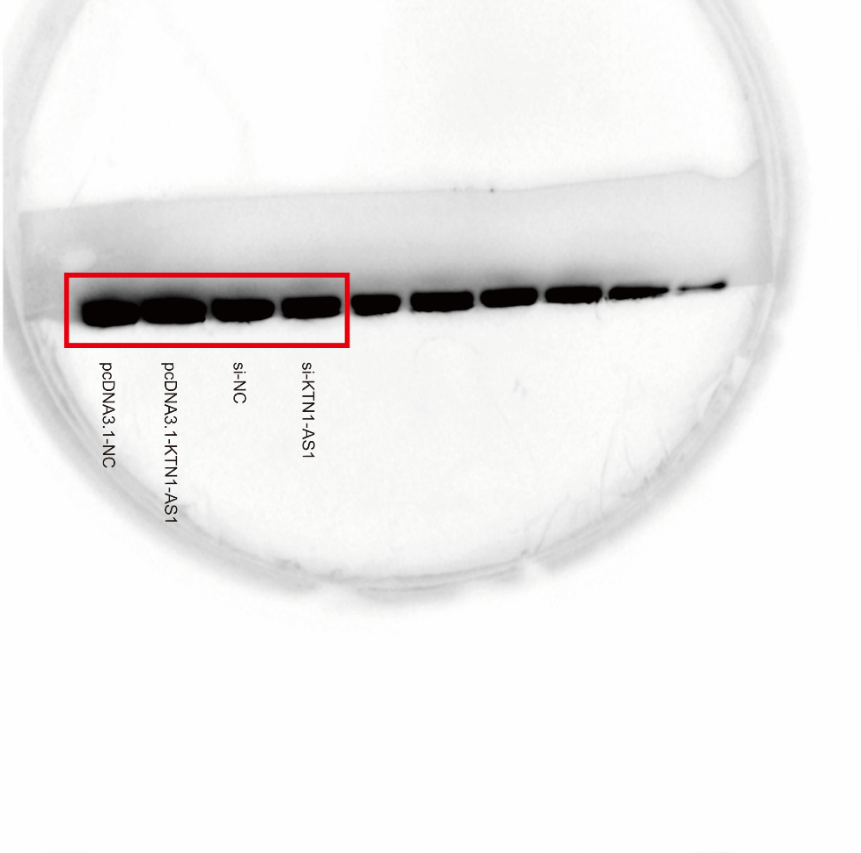


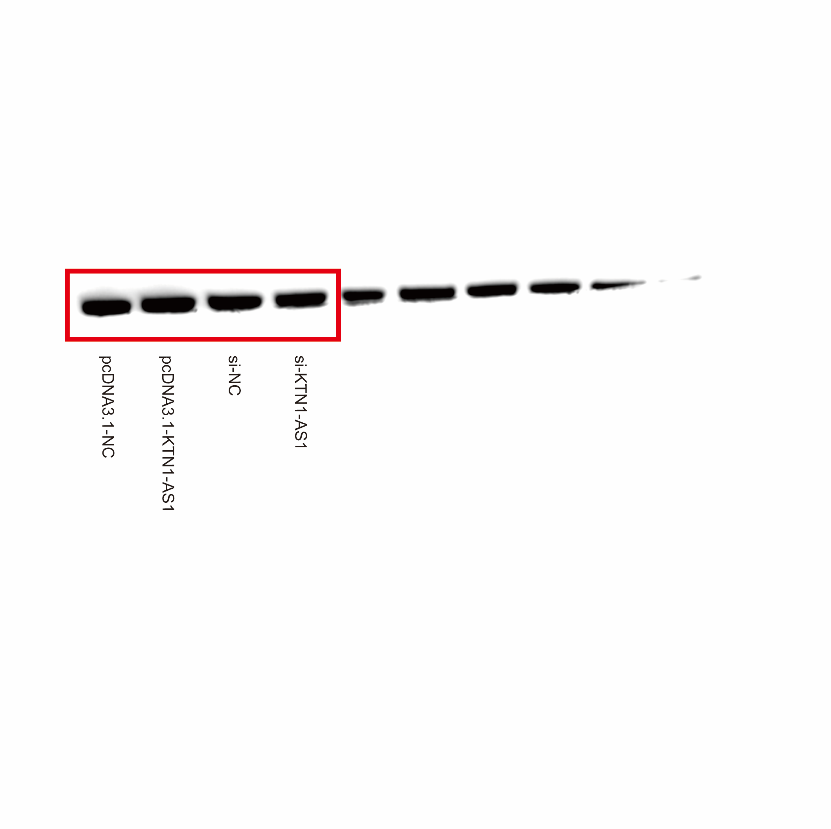


ACTB (protein bands of ACTB under different exposure intensities in Fig. 5B)

**E**


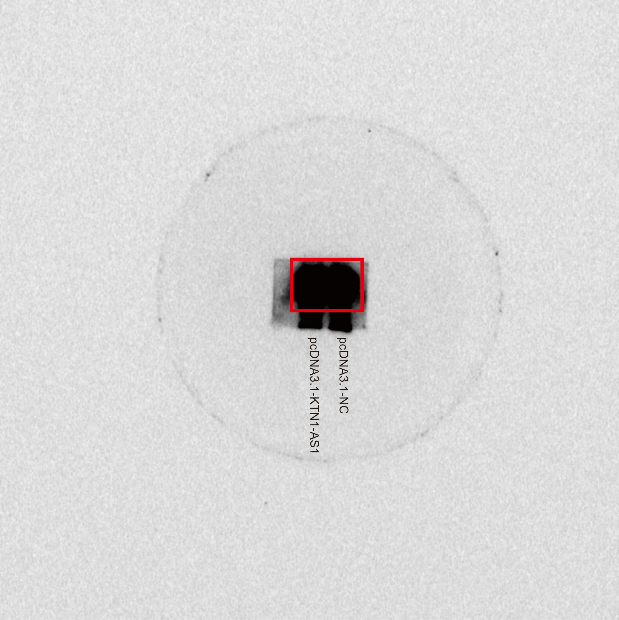

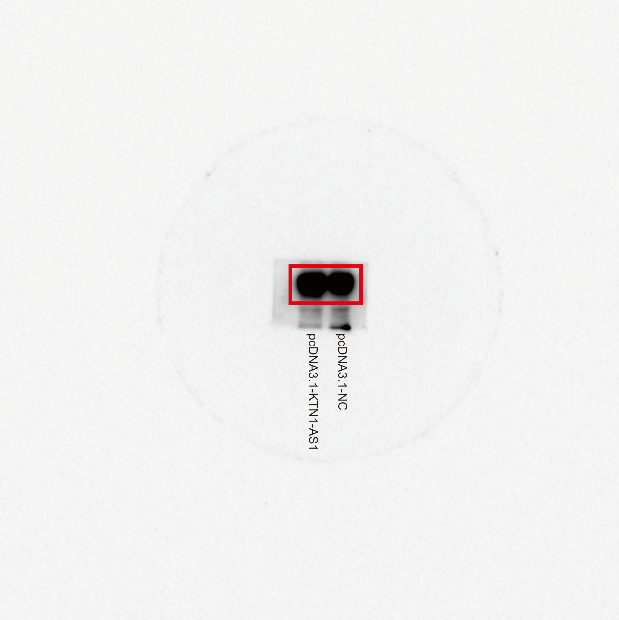


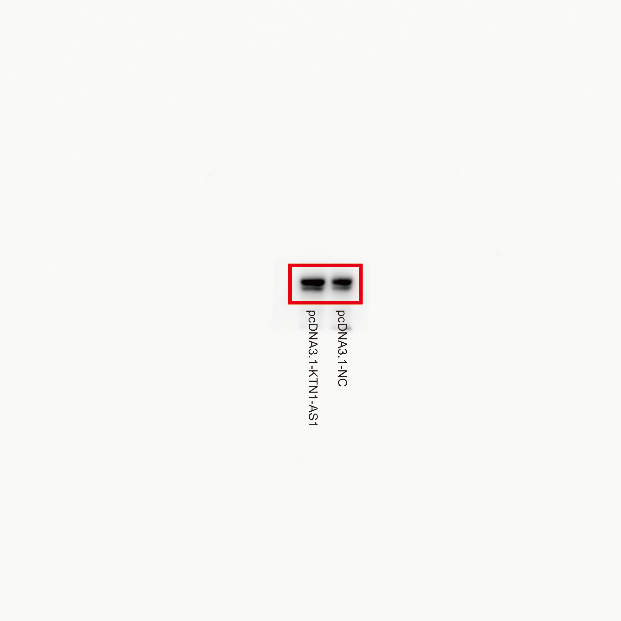

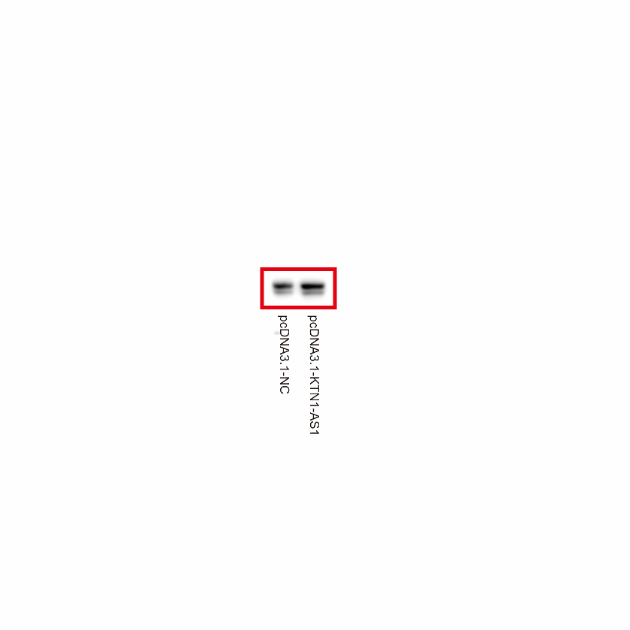


HDAC1 (protein bands of IB: HDAC1 under different exposure intensities in KTN1-AS1 overexpression group in Kyse150 cells in Fig. 5D)


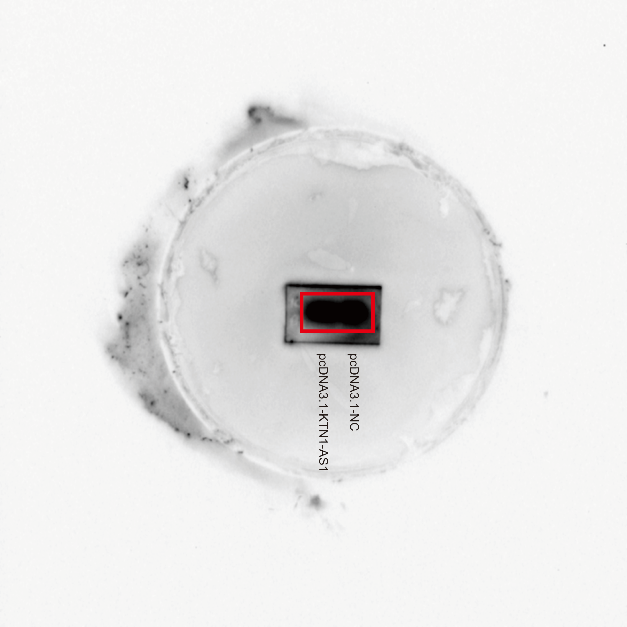

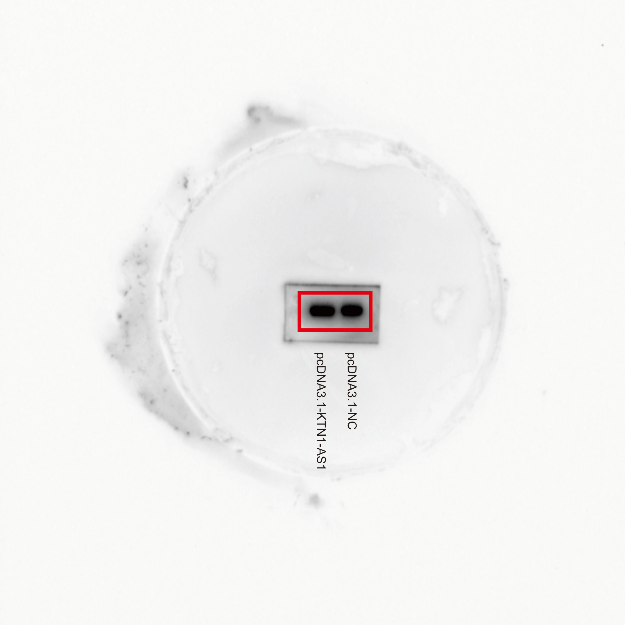


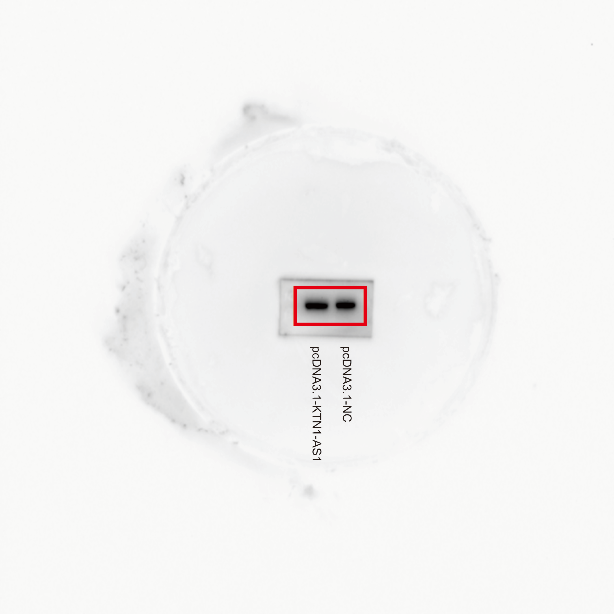

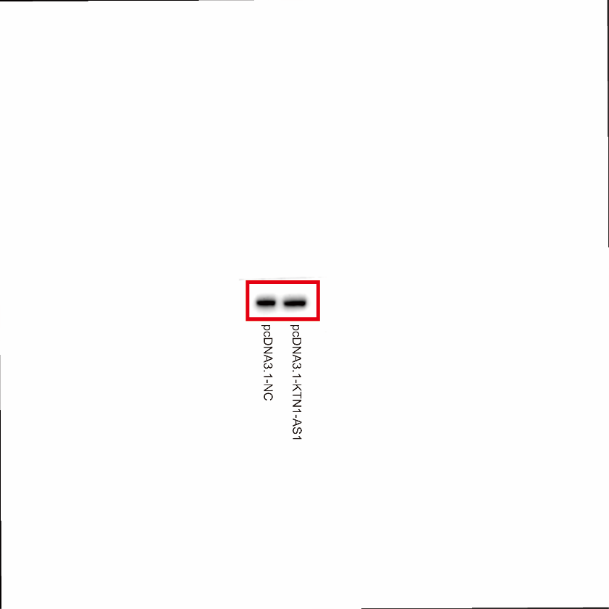


RBBP4 (protein bands of IB: RBBP4 under different exposure intensities in KTN1-AS1 overexpression group in Kyse150 cells in Fig. 5D)


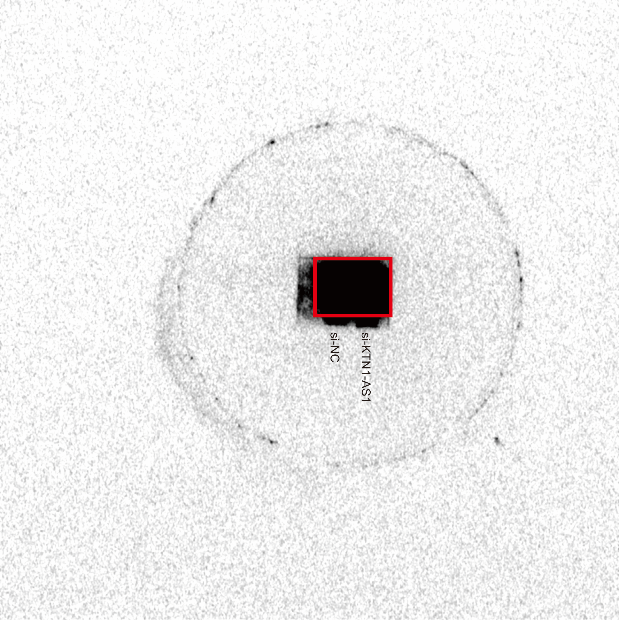

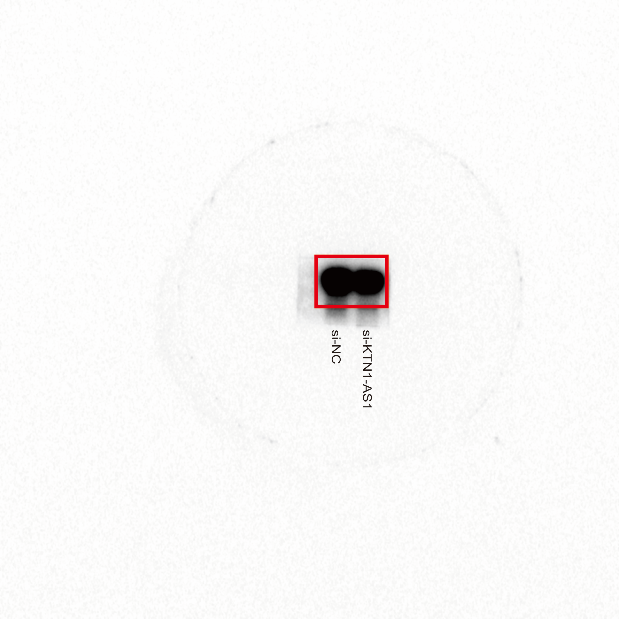


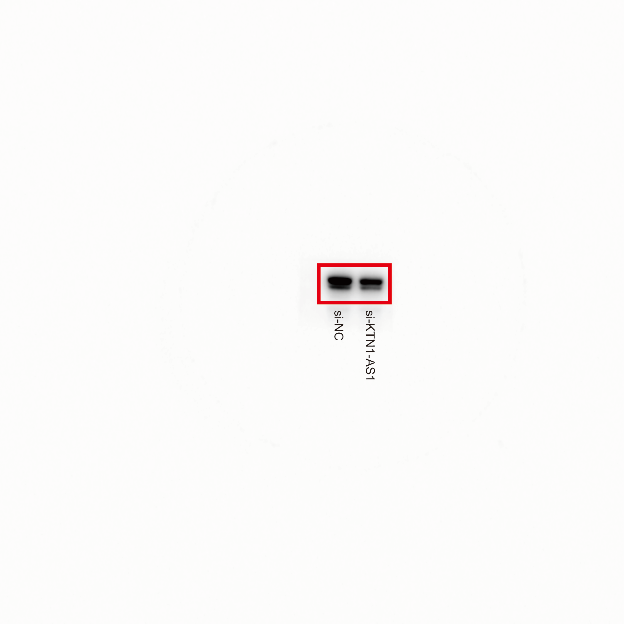

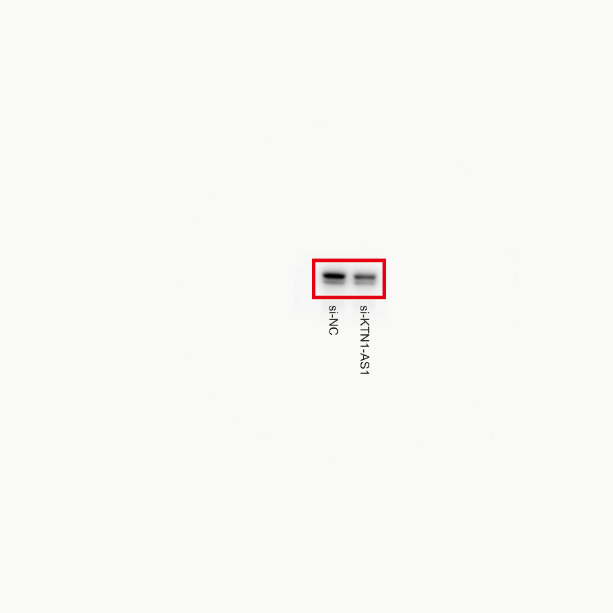


HDAC1 (protein bands of IB: HDAC1 under different exposure intensities in KTN1-AS1 knockdown group in Kyse150 cells in Fig. 5D)


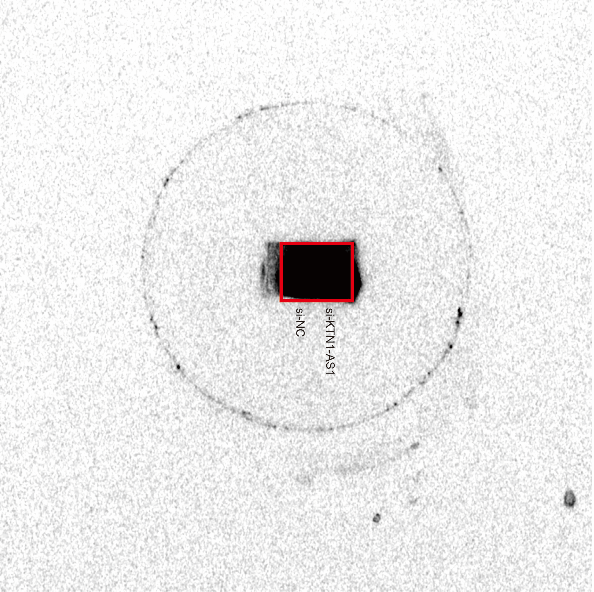

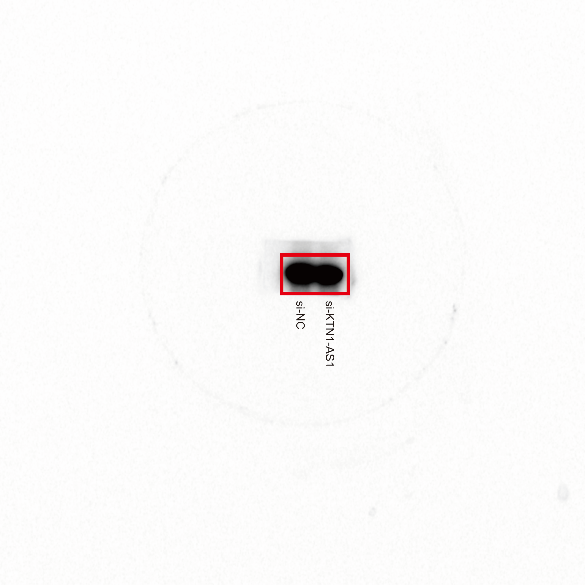

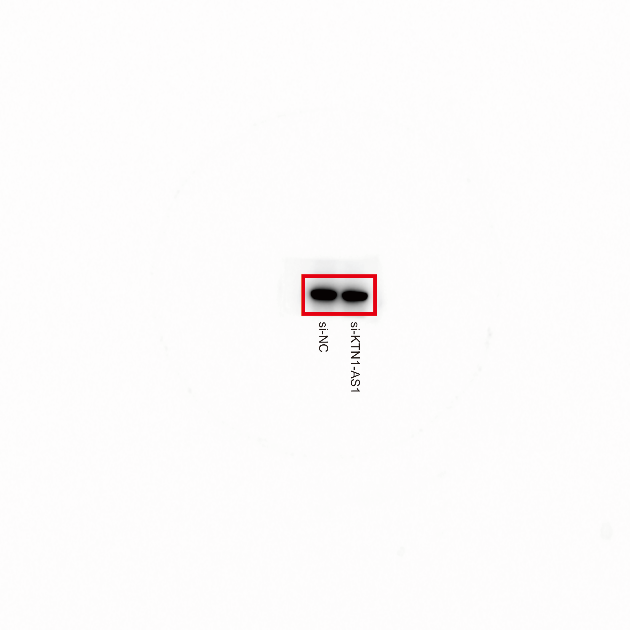

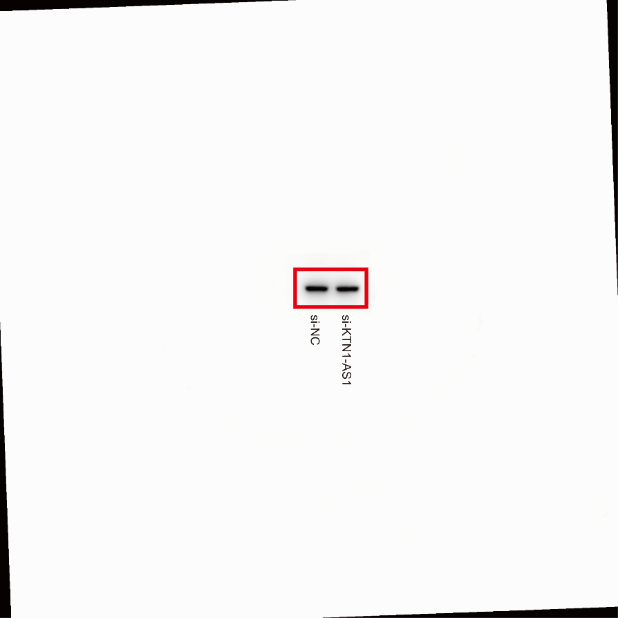


RBBP4 (protein bands of IB: RBBP4 under different exposure intensities in KTN1-AS1 knockdown group in Kyse150 cells in Fig. 5D)


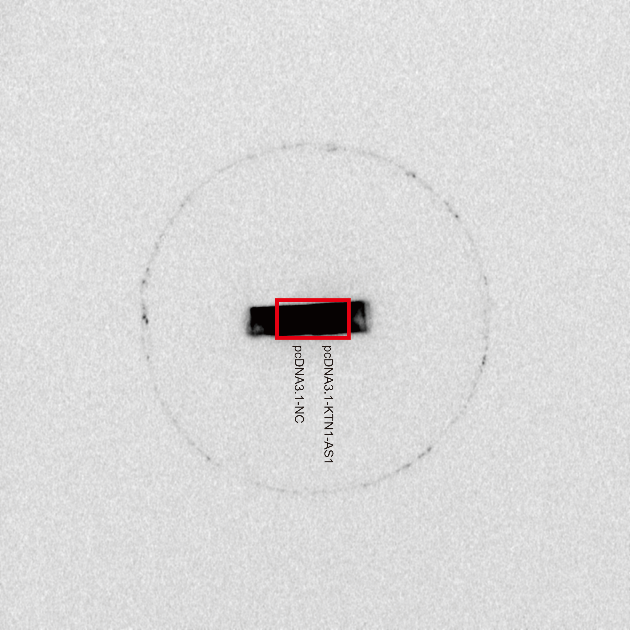

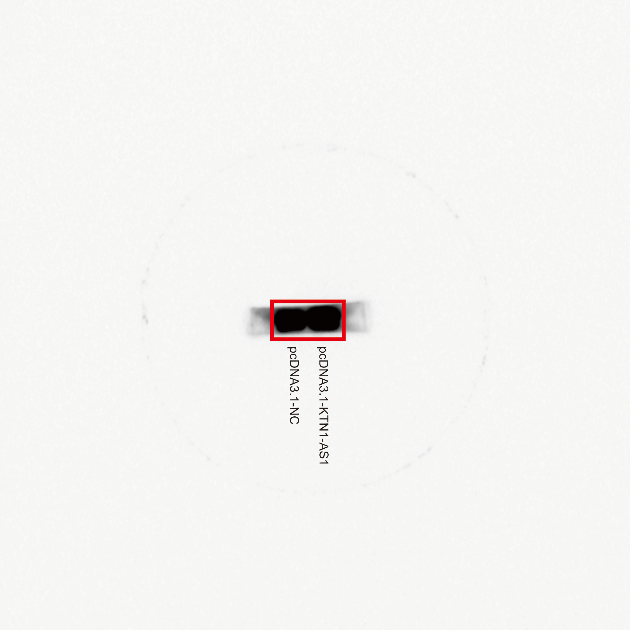


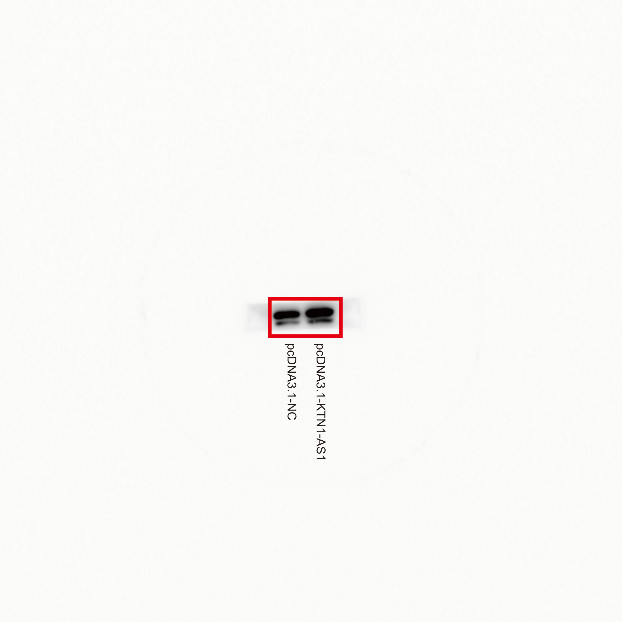

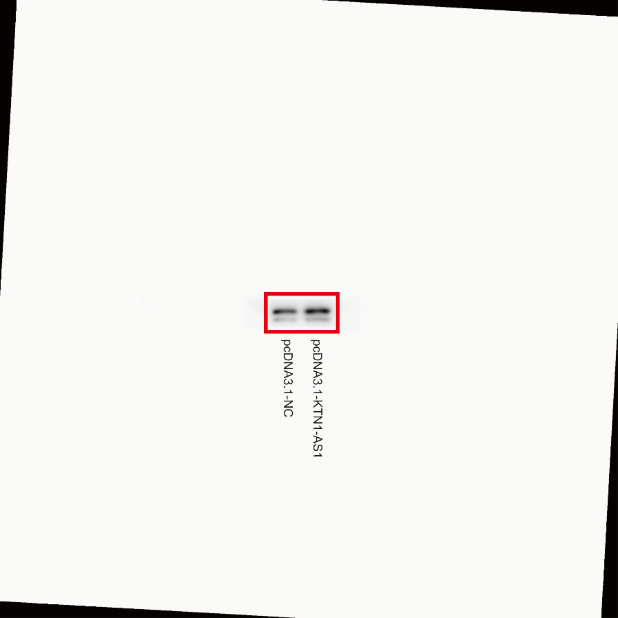


HDAC1 (protein bands of IB: HDAC1 under different exposure intensities in KTN1-AS1 overexpression group in Kyse170 cells in Fig. 5D)


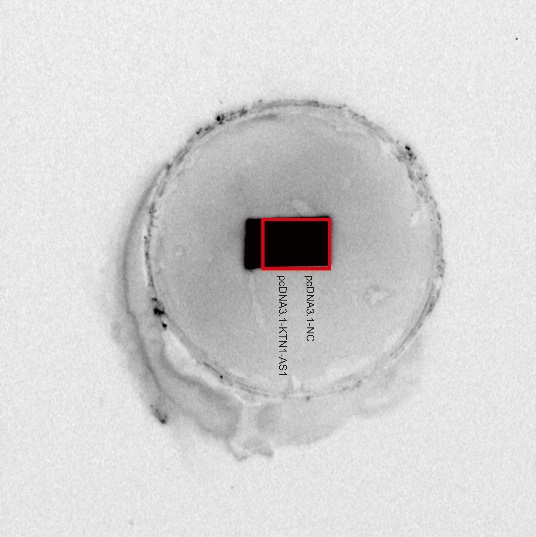

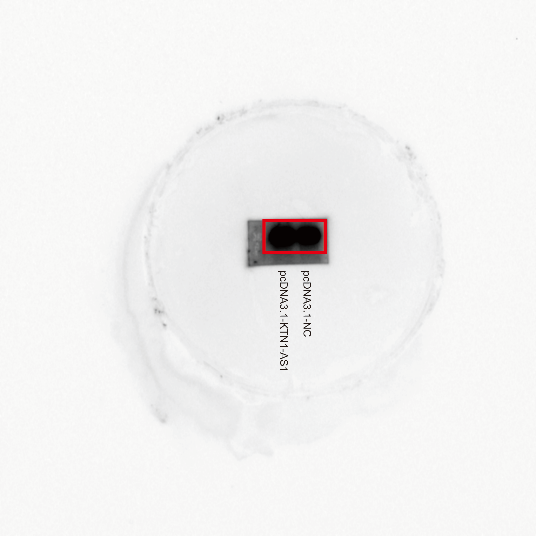


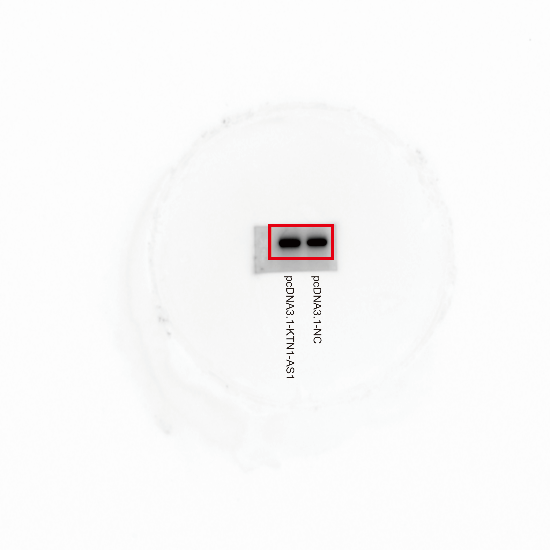

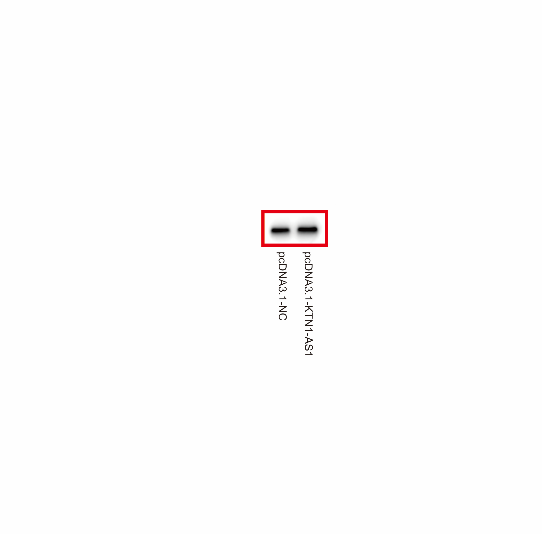


RBBP4 (protein bands of IB: RBBP4 under different exposure intensities in KTN1-AS1 overexpression group in Kyse170 cells in Fig. 5D)


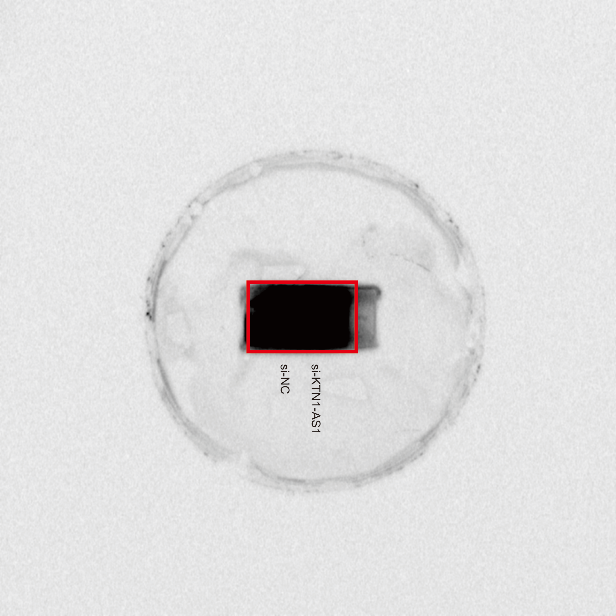

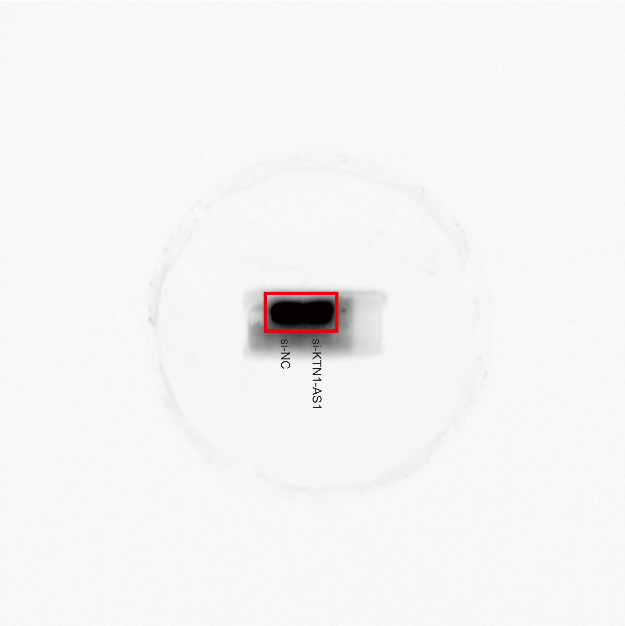

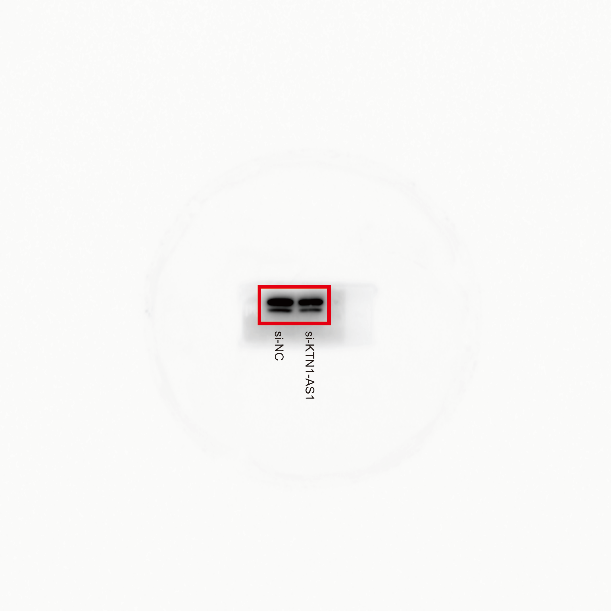

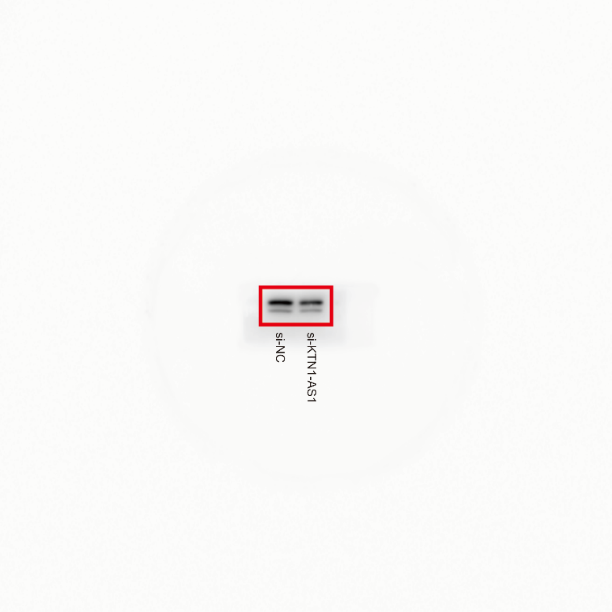


HDAC1 (protein bands of IB: HDAC1 under different exposure intensities in KTN1-AS1 knockdown group in Kyse170 cells in Fig. 5D)


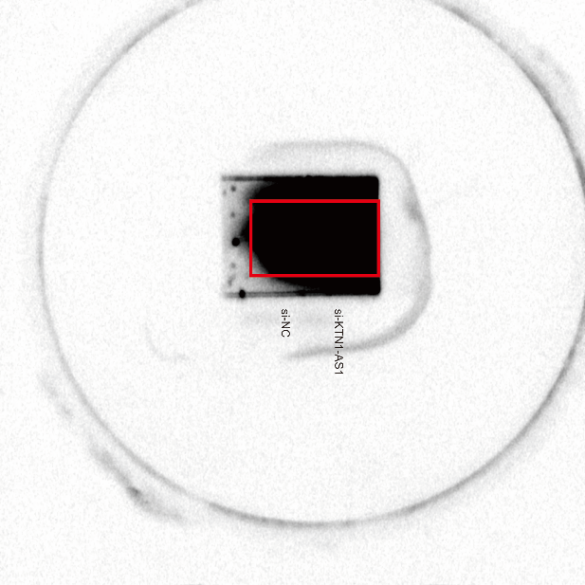

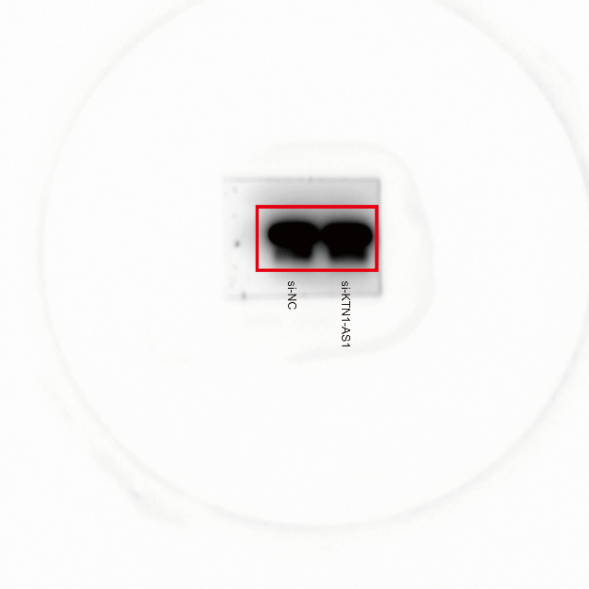


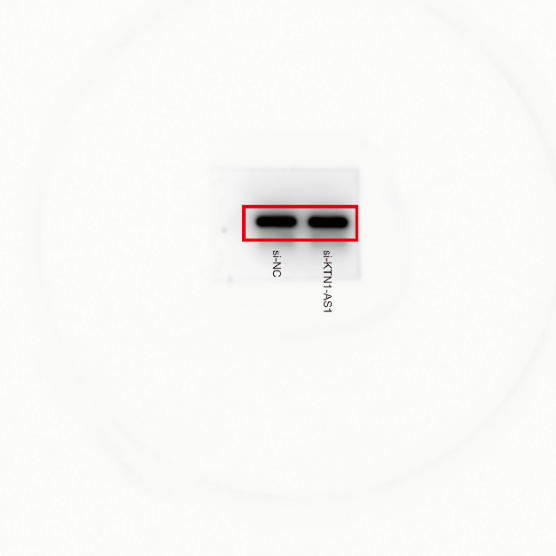

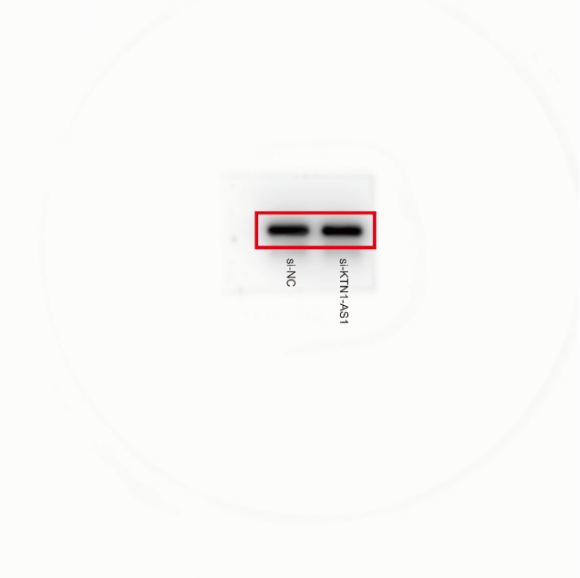


RBBP4 (protein bands of IB: RBBP4 under different exposure intensities in KTN1-AS1 knockdown group in Kyse170 cells in Fig. 5D)

**Supplementary Figure S5 The original images of the western blots in the figures**. (A) The original gel image of the pull down experiment in the figure 4B. (B) The original images of the western blots in the figure 4C. (C) The original images of the western blots in the figure 4E. (D) The original images of the western blots in the figure 5B. (E) The original images of the western blots in the figure 5D.
