# Supplementary material for: KTN1-AS1, a SOX2-mediated lncRNA, activates epithelial–mesenchymal transition process in esophageal squamous cell carcinoma
Source: Sci Rep. 2022 Nov 23;12:20186. doi: 10.1038/s41598-022-24743-z (PMC9684558; doi:10.1038/s41598-022-24743-z)
Supplement: Supplementary file 2 — Supplementary Tables. [file 41598_2022_24743_MOESM2_ESM.docx]

**Supplementary Table S1 Primers used for and qRT-PCR**

| **Names** | **Sequences** |
| --- | --- |
| KTN1-AS1 | F: 5'- TGGAGACGGAAGGTTCATCG-3'  R: 5'- GGTCAAAAGAGCACAGTGCAG-3' |
| SOX2 | F: 5'- CATGAAGGAGCACCCGGATT-3'  R: 5'- ATGTGCGCGTAACTGTCCAT-3' |
| E-cadherin | F: 5'-CGAGAGCTACACGTTCACGG-3'  R: 5'-GGCCTTTTGACTGTAATCACACC-3' |
| N-cadherin | F: 5'-CAACTTGCCAGAAAACTCCAGG-3'  R: 5'-ATGAAACCGGGCTATCTGCTC-3' |
| Vimentin | F: 5'-CGCCTGCAGGATGAGATTCAG-3'  R: 5'-TCAGGGAGGAAAAGTTTGGAAGA-3' |
| MMP2 | F: 5'-TTCCCCAAGCTCATCGCAGA-3'  R: 5'-CTCAGCAGCCTAGCCAGTCG-3' |
| U6 | F: 5'-CTCGCTTCGGCAGCACA-3'  R: 5'-AACGCTTCACGAATTTGCGT-3' |
| RBBP4 | F: 5'-GCTATGGGCTTTCTTGGA-3'  R: 5'-CACAGGCAGATGGTATGG-3' |
| GAPDH | F: 5'-AGGTGAAGGTCGGAGTCAACG-3'  R: 5'-AGGGGTCATTGATGGCAACA-3' |

F: Forward primer; R: Reverse primer.

**Supplementary Table S2**  Primers used for siRNAs

| **Names** | **Sequences** |
| --- | --- |
| si-KTN1-AS1 | F: 5'-GCUGGAGGCUCAGCGGAAUTT-3'  R: 5'-AUUCCGCUGAGCCUCCAGCTT-3' |
| si-SOX2 | F: 5'-CUGCAGUACAACUCCAUGATT-3'  R: 5'-UCAUGGAGUUGUACUGCAGTT-3' |
| si-RBBP4 | F: 5'-CCUUCUAAACCAGAUCCUUTT-3'  R: 5'-AAGGAUCUGGUUUAGAAGGTT-3' |
| si-NC | F: 5'-UUCUCCGAACGUGUCACGUTT-3'  R: 5'-ACGUGACACGUUCGGAGAATT-3' |

F: Forward primer; R: Reverse primer.

**Supplementary Table S3** Primers used for vector construction

| **Names** | **Sequences** |
| --- | --- |
| pGL3-KTN1-AS1-1 (WT)  （-1239bp ~ +56bp） | F: 5'-CCCTCGAGGAAAGAAGGGCCAACACTGAAC-3'  R: 5'-CCCAAGCTTGGAAGAAAGCCCGGAATCCC-3' |
| pGL3-KTN1-AS1-2 (WT)  （-594bp ~ +56bp） | F: 5'-CCCTCGAGCCTTCGGGAGAGTCGTCCTT-3'  R: 5'-CCCAAGCTTGGAAGAAAGCCCGGAATCCC-3' |
| pGL3-KTN1-AS1 (MUT)  （-137bp ~ -129bp） | F: 5'-CTGACGGCGTCCGGGCCCCGCG-3'  R: 5'-TGGAGTAGGCCTCGGGCATCCGGTTGCCG-3' |
| pGL3-KTN1-AS1 (MUT)  （-408bp ~ -400bp） | F: 5'-CAGACGGCGTCCGGGCCCCGCG-3'  R: 5'-ACTGATAGGCCTCGGGCATCCGGTTGCCG-3' |
| pGL3-KTN1-AS1 (MUT)  （-1113bp~-1105bp） | F: 5'-CCTACGGGGCTGCACATCCCAC-3'  R: 5'-ATAGTGGTGAAGGCGCTGGGTGC-3' |

F: Forward primer; R: Reverse primer.

**Supplementary Table S4** Primers used for ChIP

| **Names** | **Sequences** |
| --- | --- |
| KTN1-AS1-1 | F: 5'-CTGCTCTCCCTCACCCTGT-3'  R: 5'-AAGCCGCCCGTTTCCTG-3' |
| KTN1-AS1-2 | F: 5'-GTCCCGGCCCCAACAAGAG-3'  R: 5'-GGAGAGCAGGCCGCACCG-3' |
| KTN1-AS1-3 | F: 5'-AGGAATCCGTTTGCCAACACA-3'  R: 5'-CCTCGCCGGAAAACTACGC-3' |
| E-cadherin | F: 5'-GGGCTGGGATTCGAACCCAGTG-3'  R: 5'-GGGCTGGAGTCTGAACTGAC-3' |

F: Forward primer; R: Reverse primer.

**Supplementary Table S5** Results of spectrometry analysis of *KTN1-AS1* binding protein

| **Accession** | **Description** | **Gene name** | **Peptides AS-1** | **PSM AS-1** |
| --- | --- | --- | --- | --- |
| P08708 | 40S ribosomal protein S17 OS=Homo sapiens OX=9606 GN=RPS17 PE=1 SV=2 - [RS17_HUMAN] | RPS17 | 3 | 3 |
| A0A2R8Y8A0 | 40S ribosomal protein S24 (Fragment) OS=Homo sapiens OX=9606 GN=RPS24 PE=1 SV=1 - [A0A2R8Y8A0_HUMAN] | RPS24 | 2 | 2 |
| C9JPM4 | ADP-ribosylation factor 4 (Fragment) OS=Homo sapiens OX=9606 GN=ARF4 PE=1 SV=1 - [C9JPM4_HUMAN] | ARF4 | 2 | 2 |
| H0YLA2 | Signal recognition particle 14 kDa protein OS=Homo sapiens OX=9606 GN=SRP14 PE=1 SV=1 - [H0YLA2_HUMAN] | SRP14 | 2 | 2 |
| V9GYP5 | Ribosomal RNA small subunit methyltransferase NEP1 (Fragment) OS=Homo sapiens OX=9606 GN=EMG1 PE=1 SV=2 - [V9GYP5_HUMAN] | EMG1 | 3 | 3 |
| Q09028 | Histone-binding protein RBBP4 OS=Homo sapiens OX=9606 GN=RBBP4 PE=1 SV=3 - [RBBP4_HUMAN] | RBBP4 | 4 | 4 |
| P62266 | 40S ribosomal protein S23 OS=Homo sapiens OX=9606 GN=RPS23 PE=1 SV=3 - [RS23_HUMAN] | RPS23 | 2 | 2 |
| B9VP24 | 60 kDa chaperonin (Fragment) OS=Homo sapiens OX=9606 GN=HSPD1 PE=4 SV=1 - [B9VP24_HUMAN] | HSPD1 | 3 | 3 |
| O75947 | ATP synthase subunit d, mitochondrial OS=Homo sapiens OX=9606 GN=ATP5PD PE=1 SV=3 - [ATP5H_HUMAN] | ATP5PD | 2 | 2 |
| A0A3Q8ATF5 | Protein arginine N-methyltransferase 1 transcript variant 24 OS=Homo sapiens OX=9606 GN=PRMT1 PE=2 SV=1 - [A0A3Q8ATF5_HUMAN] | PRMT1 | 3 | 3 |
| D6RDA2 | Protein DEK (Fragment) OS=Homo sapiens OX=9606 GN=DEK PE=1 SV=1 - [D6RDA2_HUMAN] | DEK | 2 | 2 |
| B7Z6B3 | Receptor expression-enhancing protein OS=Homo sapiens OX=9606 PE=2 SV=1 - [B7Z6B3_HUMAN] | - | 3 | 3 |
| P49755 | Transmembrane emp24 domain-containing protein 10 OS=Homo sapiens OX=9606 GN=TMED10 PE=1 SV=2 - [TMEDA_HUMAN] | TMED10 | 2 | 2 |
| Q9H3N1 | Thioredoxin-related transmembrane protein 1 OS=Homo sapiens OX=9606 GN=TMX1 PE=1 SV=1 - [TMX1_HUMAN] | TMX1 | 2 | 2 |
| Q9Y2X3 | Nucleolar protein 58 OS=Homo sapiens OX=9606 GN=NOP58 PE=1 SV=1 - [NOP58_HUMAN] | NOP58 | 2 | 2 |
| D6RJ96 | Heat shock 70 kDa protein 4L (Fragment) OS=Homo sapiens OX=9606 GN=HSPA4L PE=1 SV=1 - [D6RJ96_HUMAN] | HSPA4L | 2 | 2 |
| Q15758 | Neutral amino acid transporter B(0) OS=Homo sapiens OX=9606 GN=SLC1A5 PE=1 SV=2 - [AAAT_HUMAN] | SLC1A5 | 2 | 2 |
| Q4ZG72 | RNA helicase (Fragment) OS=Homo sapiens OX=9606 GN=DDX18 PE=3 SV=1 - [Q4ZG72_HUMAN] | DDX18 | 2 | 2 |
| O75131 | Copine-3 OS=Homo sapiens OX=9606 GN=CPNE3 PE=1 SV=1 - [CPNE3_HUMAN] | CPNE3 | 2 | 2 |
| P13667 | Protein disulfide-isomerase A4 OS=Homo sapiens OX=9606 GN=PDIA4 PE=1 SV=2 - [PDIA4_HUMAN] | PDIA4 | 2 | 2 |
